# Constraining the timing of whole genome duplication in plant evolutionary history

**DOI:** 10.1098/rspb.2017.0912

**Published:** 2017-07-05

**Authors:** James W. Clark, Philip C. J. Donoghue

**Affiliations:** School of Earth Sciences, University of Bristol, Life Sciences Building, Tyndall Avenue, Bristol BS8 1TQ, UK

**Keywords:** genome duplication, plant evolution, polyploidy, molecular clock

## Abstract

Whole genome duplication (WGD) has occurred in many lineages within the tree of life and is invariably invoked as causal to evolutionary innovation, increased diversity, and extinction resistance. Testing such hypotheses is problematic, not least since the timing of WGD events has proven hard to constrain. Here we show that WGD events can be dated through molecular clock analysis of concatenated gene families, calibrated using fossil evidence for the ages of species divergences that bracket WGD events. We apply this approach to dating the two major genome duplication events shared by all seed plants (*ζ*) and flowering plants (*ɛ*), estimating the seed plant WGD event at 399–381 Ma, and the angiosperm WGD event at 319–297 Ma. These events thus took place early in the stem of both lineages, precluding hypotheses of WGD conferring extinction resistance, driving dramatic increases in innovation and diversity, but corroborating and qualifying the more permissive hypothesis of a ‘lag-time’ in realizing the effects of WGD in plant evolution.

## 1. Background

The discovery in plant genomes of evidence of recurrent whole genome duplication events (WGD; polyploidy) has reignited debate over its importance in land plant evolution [1,2]. Several causal hypotheses have emerged linking WGD to key innovations [3], increased rates of diversification [4] and extinction resistance that may have facilitated the success of multiple lineages of extant plants [5]. The mechanisms through which genome duplication can result in evolutionary novelty are becoming better understood and the traditional models of neo- and subfunctionalization have now been hybridized with models of dosage balance in attempts to explain how evolutionary innovation can arise post-WGD in the face of extensive gene loss and stabilizing patterns of gene retention [6,7]. Furthermore, there now exist elegant examples of genes and gene families that have taken on new functions (neofunctionalization) following multiple rounds of WGD and then playing a key role in the evolution of plant lineages [8]. The link between polyploidy and diversification remains controversial [9], but there exists some evidence that several of the ancient WGD events in angiosperms correlate with shifts in diversification [4]. Separating the WGD events and the shifts in diversification are a ‘lag’ of several million years, which has been explained as the period of fractionation post-WGD and, in turn, the feature of WGD that leads to innovation and diversification [10]. However, at the broadest scale, these hypotheses are underpinned by the relative phylogenetic placement and absolute timing of each event. Though the relative phylogenetic timing of plant WGD events is well constrained, their absolute timing is not [9].

Constraining the phylogenetic position of WGD events relies on broad taxonomic sampling of genomic or transcriptomic data. The presence or absence of shared ‘age peaks’ in Ks plots of synonymous substitution rates between duplicates provides evidence for shared genome duplications [11]. This approach culminated in a survey of 41 plant genomes focusing on angiosperms [5] and more recently several transcriptomes also highlighting the presence of WGD within the evolutionary history of gymnosperms [12] and peat mosses [13]. The number and position of the peaks on the Ks plot also reveals the relative timing of each event, with multiple peaks representing multiple successive WGDs. The absolute timing of each event can be obtained indirectly by phylogenetically bracketing the event—the event must have occurred along the branch between those lineages that have undergone the WGD and those that have not. However, despite well-sampled exceptions among certain groups of angiosperms [14–16], there are few cases where the sampling of taxa is dense enough to prevent very long branches, and so the ages of genome duplication events must be inferred directly. Direct dates can be obtained by converting the relative timing of peaks on a Ks plot into absolute ages. This has the advantage that it does not require additional taxon sampling and so estimates can be obtained for WGD events isolated on long branches [17]. A major caveat of this approach is that it relies on the assumption of a strict molecular clock that, depending on shifts in the rate of sequence evolution, can lead to inaccurate age estimates. Furthermore, Ks plots are known to saturate beyond a certain age, meaning that they cannot always distinguish more ancient duplications and may lead to artificial peaks in the distribution [18]. More complex relaxed clock methods can be employed in a phylogenetic or phylogenomic approach, whereby the individual gene families containing signal of WGD are reconstructed and individually dated [19]. The distribution of ages obtained can then be plotted to provide a range of estimates for each event. This approach is more powerful and has been used to estimate the ages of multiple WGD events across the angiosperms, where genomic and transcriptomic data are more abundant [19,20]. However, dating individual gene trees does not fully exploit the power of the molecular clock and the power of individual gene trees is likely to diminish over longer periods of evolutionary time. Increasing the amount of sequence data by concatenating multiple gene families into alignments decreases uncertainty in the estimation of relative ages [21], and can be used to date the absolute timing of WGD events [22] yet, to date, studies focusing on WGD in plants have relied on the power of individual gene trees. Directly dating WGD events using concatenated gene trees also provides estimates of the absolute timing of the WGD in relation to subsequent speciation events within the lineage, since gene trees observe species divergences as well as duplication events. Thus, concatenated gene trees have the potential to provide an accurate estimate of the absolute timing of WGD events relative to the diversification events in which they are causally implicated.

The seed plants (Spermatophyta) are the most species rich of extant plant clades, encompassing the gymnosperms and angiosperms (flowering plants). WGD events have been identified at the base of all seed plants (*ζ*; [12,20]) and at the base of all angiosperms (*ɛ*; [20]), and so all extant flowering plants have undergone at least two rounds of genome duplication. Previous attempts to date these events were based on distributions of ages inferred using poorly defined calibrations and penalized likelihood molecular clock methods [20] that have since been found unreliable [23]. The WGD shared by all extant angiosperms has been linked with the ‘big bang’ diversification of the Mesangiospermae (following a lag period) as well as several major innovations, including the origin of the flower [3,4]. WGD has been thought to be less prevalent within gymnosperms, the sister clade to angiosperms (together comprising Spermatophyta), despite the fact that the *ζ* WGD is part of their shared evolutionary history. More recent evidence has indicated that WGD has occurred in several gymnosperm lineages and confirmed that the *ζ* WGD (spermatophyte) was not shared with their sister lineage, the ferns [12].

Conventionally molecular clock dating approaches have sought to minimize the influence of duplication by using only single copy genes. In contrast, we exploit the pattern of paralogy produced by WGD in the evolutionary history of multiple gene families and concatenate them into a partitioned alignment. Combined with broad taxon sampling and multiple fossil calibrations, we demonstrate an approach for dating gene trees to provide well-constrained estimates of the timing of duplication events and attendant speciation events.

## 2. Material and methods

Gene families containing signal of the *ζ* (spermatophyte) and *ɛ* (angiosperm) WGD events and those that contain the signal of both were catalogued by Jiao *et al*. [4], and from these we expanded orthogroups by obtaining amino acid sequences using Plaza 3.0 (bioinformatics.psb.ugent.be/plaza), and GreenPhyl 4 (www.greenphyl.org). Further sequences were obtained by local BLAST searches of iPlant (www.iplantcollaborative.org). One hundred and twenty-eight species were sampled in total, representing all major lineages of land plants and these are listed in electronic supplementary material, table S1. Four datasets were assembled for all taxa: families containing a clear signal of just the *ɛ* WGD event (angiosperm dataset), just the *ζ* WGD event (spermatophyte dataset), families containing signal of both events (*ζ* + *ɛ* dataset), and a combined dataset. To verify a clear signal of the relevant WGD event in each gene family, we built individual gene trees based on multiple amino acid sequence alignments generated using MAFFT while model selection and gene tree reconstructions were performed using IQ-TREE [24]. We opted for a conservative approach, discarding orthogroups that following phylogenetic reconstruction and visual inspection did not clearly reflect the signal of either or both WGDs (e.g. electronic supplementary material, figure S1), had sequence alignments shorter than 100 amino acids, displayed a topology that was incongruent with our current understanding of land plant phylogeny with either the total group seed plants or major lineages within being resolved as non-monophyletic, or were too large with multiple nested duplications, resulting in large numbers of sequences having to be discarded. Of 130 orthogroups surveyed, 12 gene families were found containing a clear signal of the *ɛ* WGD. The number of sequences among individual gene families ranged from 87–126 and when concatenated a total of 176 tips. Fourteen further gene families were found for the *ζ* WGD, representing 189 tips when concatenated and varying from 106 to 149 tips individually. An additional seven gene families were found containing the signal for both, for which 254 tip sequences were assembled when concatenated and individual gene families ranging from 132 to 249 tips. The combined dataset contained 33 gene families, with one node representing *ζ*, but two representing *ɛ*. As 12 gene families contain only one node with the *ɛ* duplication, the event was represented only once in the combined analysis, to maximize precision at this node. Similarly, angiosperm gene copies from gene families not containing signal of the *ɛ* duplication were randomly assigned to one side of the duplication. Due to differential retention, a copy of each gene paralogue was not present in all families and the number of tips in each gene family is listed in electronic supplementary material, table S3.

**Figure 1.**
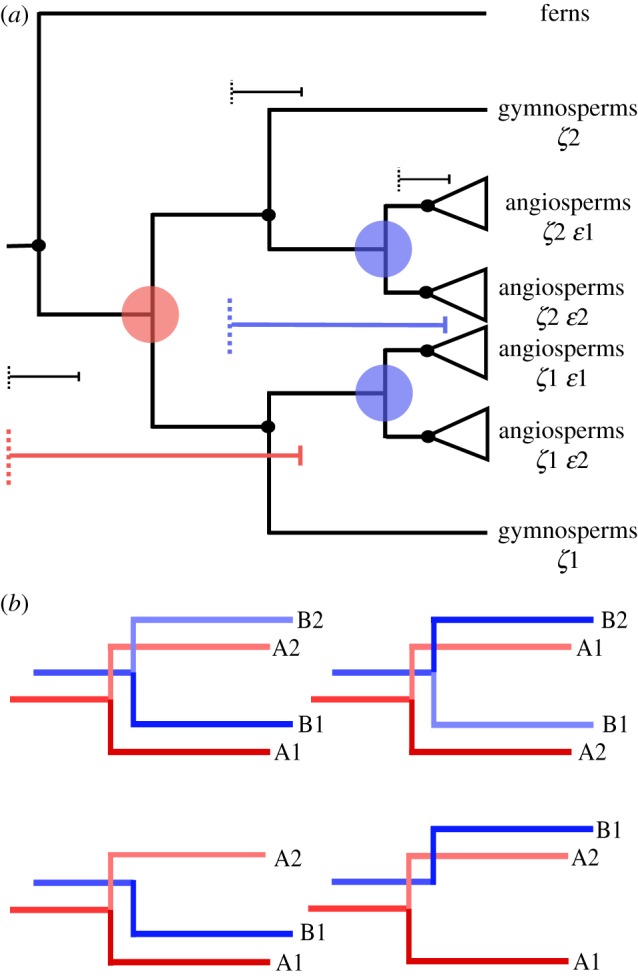
(*a*) An example gene tree showing the seed plant (*ζ*, red) and angiosperm (*ɛ*, blue) duplications. The duplication events are constrained using minima and maxima (coloured brackets) based on fossils used to constrain speciation events (black brackets). (*b*) Gene trees may retain both copies of the duplicate gene (top), or a single copy may be lost (bottom). When concatenating duplicates from different gene families, given that both copies are descended from the same event, their assignment to either side of the duplication is arbitrary. (Online version in colour.)

Across all analyses, nodes were constrained using 35 fossil calibrations spanning land plant phylogeny defined using best practice [25] (electronic supplementary material, table S2). The duplication nodes were constrained temporally to reflect the possibility of the WGD occurring at any point following the divergence of spermatophytes from an ancestral euphyllophyte (*ζ* WGD event) and for angiosperms from an ancestral spermatophyte (*ɛ* WGD event) (figure 1). Calibrations that provided only a minimum age were modelled as a hard minimum bound with a truncated Cauchy distribution (*p* = 0.1, *c* = 0.2). Calibrations that provided a maximum age were modelled with a soft maximum with a uniform distribution between the minimum and maximum age [26]. Molecular clock analyses were conducted on concatenated alignments using the normal approximation method in MCMCtree under the appropriate model [27]. The normal approximation method provides a fast and efficient way of analysing large datasets using complex models and a relaxed clock and is run under a fixed topology. We ran all analyses on a topology reflecting both WGD events and recent hypotheses of relationships among land plants [28] (electronic supplementary material, figure S2). We also reconstructed the topology based on our own datasets using IQ-TREE and found that it was highly congruent with the constraint tree. Each analysis was run twice independently and regularly checked for convergence and for effective sample sizes greater than 200 using Tracer v. 16 [29].

Assuming autopolyploidy, each WGD event produces two daughter nodes that are created simultaneously and that must have the same age, and so the assignment of each paralogue to either node of the duplication is arbitrary (figure 1). In this way paralogues between the gene families can be concatenated in multiple combinations, so long as they are consistent within each gene family. To explore the impact of different combinations of paralogy groups between gene families, we randomly reassigned groups to either node using the *ζ* + *ɛ* dataset containing both duplications.

The extent to which the low number of available gene families impacted on the estimation of dates was explored through infinite sites analyses [30]. The gene families were successively concatenated and the analysis repeated with one more gene family each time. The relationship between the mean age estimates and the widths of the 95% HPDs was used as a measure of the precision of the data versus the uncertainties induced by the fossil calibrations. Higher *R*^2^ values indicate that large HPD widths are due to increasing uncertainty in the fossil record deeper in time. A saturation of the curve suggests that adding further sequence data would not increase the precision of the analysis, since it is limited by the information available in the fossil record.

## 3. Results

In most Bayesian molecular software, specified node age priors are modified in the construction of the joint time prior to achieve the expectation that only ages compatible with the assumption that ancestral nodes are older than their descendants, are proposed to the MCMC [31,32]. To ensure that these effective priors are biologically reasonable, we estimated them by running the analysis without sequence data. The effective priors are compatible with the original palaeontological and phylogenetic evidence, yielding broad 95% HPDs for the timing of WGDs in all analyses, though both were truncated relative to the specified calibrations. The spans of the 95% HPD for the prior on the *ζ* and *ɛ* WGD events are 81 (434–353 Ma) and 111 (355–244 Ma) million years, respectively (table 1). In the separate analyses of both the *ζ* and the *ɛ* WGD events, the truncation effects on the prior were the same as for the combined analysis, and so the additional nodes in the combined analysis and the *ζ* + *ɛ* dataset did not affect the effective prior.

**Table 1.** Ninety-five per cent HPD estimates for the age of both WGD events, summarizing the effective prior, individual gene families (1 to 7), the effects of concatenating gene families, the expanded and combined datasets.

node	effective prior	gene families							concatenated gene families						*ɛ* dataset	*ζ* dataset	combined dataset
		1	2	3	4	5	6	7	1–2	1–3	1–4	1–5	1–6	1–7			
spermatophyte duplication (*ζ*)	353–434	382–435	346–411	346–411	354–418	354–404	357–415	355–433	390–433	386–430	380–418	380–416	377–408	378–409	—	380–401	381–399
angiosperm duplication (*ɛ*) *ζ*1 *ɛ*	244–355	270–339	250–353	248–328	280–354	258–340	249–351	254–356	273–336	268–323	280–323	285–331	282–325	281–323	295–321	—	297–319
angiosperm duplication (*ɛ*) *ζ*2 *ɛ*	244–355	267–340	273–344	247–350	245–349	277–362	247–313	245–355	278–333	276–330	276–322	289–338	283–325	276–321	—	—	—

In all instances, the addition of sequence data yielded estimates congruent with, yet more precise than, the joint time prior. Estimates for both WGD events were compared between gene families using the *ζ* + *ɛ* dataset, and we found variation in both the width of the 95% HPD and the absolute age estimates, though the overlapping distributions of the HPDs showed that the gene families were congruent. While some gene families produced much more precise estimates, the variation in estimates between all gene families showed a similar level of precision to the joint time prior alone, ranging from 435–346 Ma for the *ζ* WGD event and 355–244 for the *ɛ* WGD event. The *ζ* + *ɛ* dataset also allowed us to compare the estimates for the *ɛ* duplication, which is represented twice in each gene family, within gene families. We found that the 95% HPD widths for the event varied within gene families, though this is likely due to the absence of paralogues on one side of the duplication. The only family with all paralogues present, CDK, showed estimates consistent in both age and uncertainty across both nodes.

The greatest effect in terms of precision was produced by increasing the amount of sequence data by concatenating the gene families. The effect of missing paralogues across both duplication nodes in the *ζ* + *ɛ* dataset was minimized and the age estimates for both *ɛ* nodes were highly consistent. The *ζ* + *ɛ* concatenation was also considerably more precise than any of the individual gene families (table 1). Multiple concatenations were tested on this dataset, to determine if the assignment of paralogues between duplicates affected the estimates. We did not observe any material differences in age or uncertainty, indicating that the results are robust to the way in which the gene families are concatenated.

**Figure 2.**
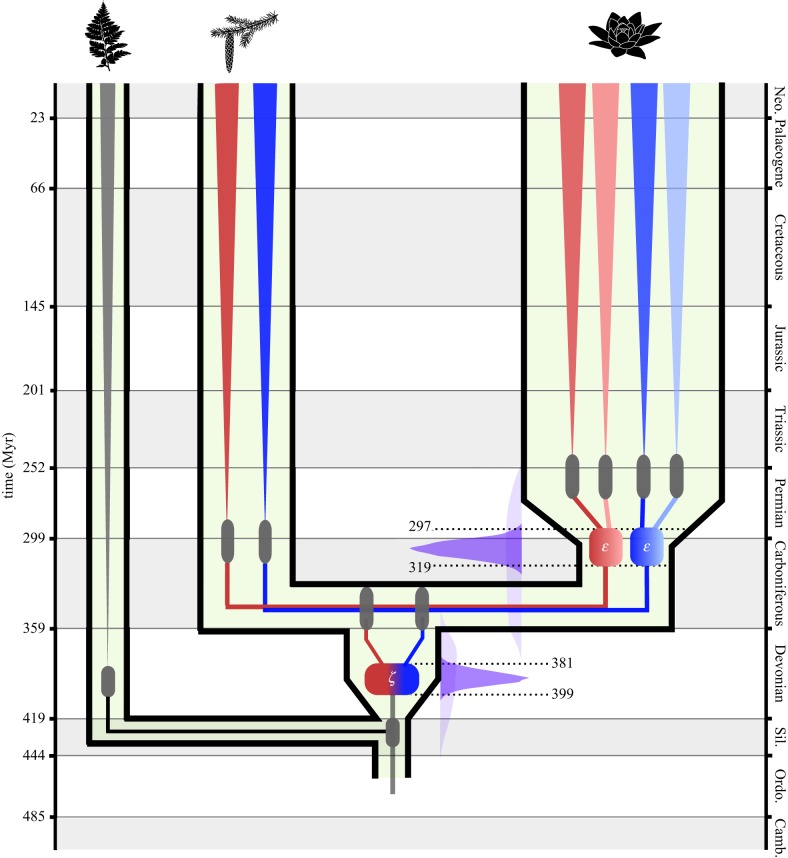
Estimated dates for the occurrence of both the seed plant (*ζ*) and angiosperm (*ɛ*) duplication events based on a molecular clock analysis of 33 concatenated gene families. Age estimates (95% HPD) for the divergences of the major lineages and crown groups represented by grey bars. The age estimates (95% HPD) of two duplication events are represented by coloured boxes, with the subsequent subgenomes represented first by blue and red (*ζ*), then by lighter and darker shades of each colour (*ɛ*). For each duplication event, the effective prior is shown (light blue) next to the posterior distribution (dark blue). (Online version in colour.)

The addition of further sequence data for each duplication event in turn produced results of even greater precision. The angiosperm dataset estimated an age of 321–295 Ma for the *ɛ* WGD event, almost five times more precise than the joint time prior alone. A similar increase in precision was obtained by the spermatophyte dataset, the *ζ* duplication estimated to have occurred 400–380 Ma, four times more precise than the joint time prior alone. Based on the largest amount of data, the combined analysis of the combined dataset produced results that were highly congruent with the two individual datasets, if not marginally more precise, estimating 399–381 Ma and 319–297 Ma for the *ζ* and *ɛ* WGD events, respectively (figure 2).

**Figure 3.**
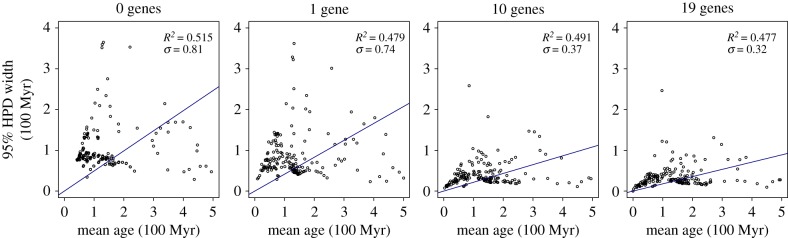
Infinite sites plots for the most complete (angiosperm) dataset, with the regression between the mean age and the 95% HPD shown for 0, 1, 10 and 19 gene datasets. The *R*^2^ and error terms are also shown. (Online version in colour.)

Infinite sites plots suggest that though the *R*^2^ value showed little changed with increased sequence data, the addition of sequence data reduced the uncertainty of estimates (figure 3). With 19 gene families, the amount of error was continuing to decrease, suggesting that additional gene families may increase precision further.

## 4. Discussion

### (a) Inferring the age of whole genome duplication

Our results indicate that the evolutionary history of gene families can be exploited to obtain precise estimates of the age of WGD events. These methods depend on both careful selection of fossil constraints and available gene families containing signal of WGD events, though even with limited sequence data, we greatly improve the precision over the raw calibrations alone.

Both the *ɛ* (angiosperm) and *ζ* (spermatophyte) genome duplication events have been independently reported [12,20], yet we were unable to find large numbers of gene families with clear signal of either or both events. The paucity of available gene families for these WGD events is likely in part a result of our conservative criteria in selecting gene families based on topology. In part, this reflects the limitations of single genes to resolve unequivocal phylogenetic signal for such events over long timescales. However, it also reflects the antiquity of the events, given that retention of genes following a WGD follows a decay pattern and widespread gene loss leads to a gradually decreasing phylogenetic signal over time. It is unsurprising that so few gene families remain with a clear signal of these events and, when considered next to existing evidence for these events [12,20], our findings are entirely compatible with the *ɛ* and *ζ* duplication events. Our results indicate that the evolutionary history of gene families can be exploited to obtain precise estimates of the age of WGD events. Infinite sites plots lead us to expect that the addition of further sequence data will leverage further precision. Similarly, WGD events that are more recent and may contain more genome-wide data, may be dated using the same approach but with greater precision.

Unlike genomic datasets that can be used for gene-tree reconciliation and the construction of Ks plots, the methods presented here focus solely on the dating of WGD events, rather than their characterization. However, the congruence of age estimates between gene families serves as a test of their coincidence, as anticipated by WGD. The annotation of gene families to either side of the duplication event requires greater care and is a potentially limiting factor on the number of gene families that can be analysed, yet we have demonstrated that even with a relatively small dataset (compared to a genomic dataset), high levels of precision can be achieved. Novel molecular clock approaches such as cross bracing could also be used to increase precision around the duplication nodes, especially as they are so difficult to constrain [33].

An additional caveat is that WGD or polyploidy is often categorized into two distinct classes [34], autopolyploidy and allopolyploidy, traditionally distinguished based on the number of parent species, but also characterized by the patterns of fractionation post-WGD. The mode of duplication may impact our estimates of duplication age [35], as the point at which duplicates coalesce is actually the timing of divergence of the two parental species, or a more ancestral autoploidy event, as opposed to the alloploidy event itself [35]. New methods are emerging to discriminate between auto- and allopolyploidy [36], but these are likely to fail when applied to more ancient genome duplication events. However, allopolyploidy would only have a large impact on accuracy if hybridization occurred between very distant parent species.

### (b) Dating duplication, diversification and innovation

**Figure 4.**
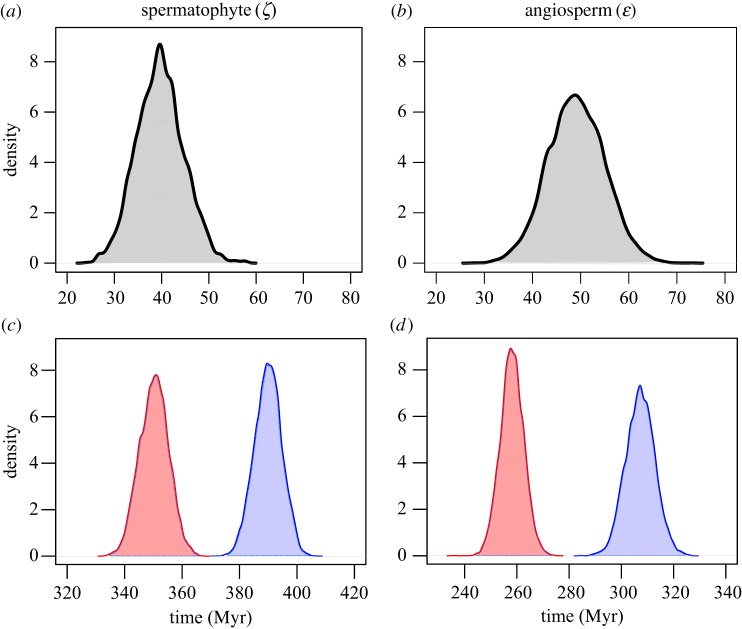
The posterior probabilities of (*a*) the lag between the *ζ* duplication and the diversification of crown spermatophytes and (*b*) the lag between the *ɛ* duplication and the diversification of crown angiosperms. The posterior probabilities of the absolute age of the WGD events (blue) and diversification (red) are also shown for (*c*) *ζ* and spermatophytes and (*d*) *ɛ* and angiosperms. (Online version in colour.)

Our most comprehensive analysis of 33 gene families indicated that the genome duplication present in all crown spermatophytes occurred 399–381 Ma, a period spanning the Early to Late Devonian (figure 2). The WGD event present in all crown angiosperms occurred almost 100 Myr later, 319–297 Ma, across the Carboniferous–Permian boundary (figure 2). Gene trees contain both the signal of WGD and species divergence, allow a direct estimation of the age of the WGD event relative to the age of the crown group (figure 4). Both estimates predict that the respective WGD events occurred early in the stem of both lineages, predating the diversification of the crown group by about 50 Myr. These estimates are considerably older than those of Jiao *et al*. [20], yet our estimates for the age of the seed plant (360–340 Ma) and angiosperm (267–247) crown groups are comparable to other molecular clock analyses [37,38], allowing us to reject the notion that the duplications occurred late in the stem lineage. Greater precision in the absolute age of WGD events leveraged by concatenation allows that hypotheses can be more rigorously tested. WGD occurring early in the stem lineage has two implications for current hypotheses regarding the role of WGD in plant evolution.

First is the hypothesis that WGD drives evolutionary success [39–41], or confers extinction resistance [19,42], since the long stem lineages of both groups are, by definition, characterized by extinction. However, many extinct lineages must also share these genome duplications. For example, the *ζ* duplication predates the appearance of the earliest seed plants, the pteridosperms and cordaitales, and so WGD cannot have contributed to their diversification or conferred extinction resistance, as has been proposed for the ancient palaeopolyploid *Equisetum* [17]. The long-term evolutionary success of seed plants and especially angiosperms is unquestionable, and there is considerable evidence for the role of gene duplication in the evolution of angiosperms, in particular [3,43], yet our results are more in keeping with the idea of ‘rarely successful polyploids’ [39]. The challenges faced by polyploids in order to establish and persist may be partially responsible for extinctions in a lineage post-WGD, and it may be the case that extant spermatophytes and angiosperms are the surviving lineages best able to exploit any long-term competitive advantages [42]. Secondly, if their crown clades of seed and flowering plants can be considered to be characterized by evolutionary success, this has been achieved in both lineages after a substantial lag post-WGD. Our results indicate that the lag between the *ζ* WGD event and the divergence of crown spermatophytes is 22–60 Myr, and 27–65 Myr between the *ɛ* WGD event and the divergence of crown angiosperms (figure 4). These are comparable to the results of Tank *et al*. [4], who estimated a 49.2 Myr lag between the *ɛ* WGD event and the shift in diversification of angiosperms, though without directly inferring the age of the WGD. Tank *et al*. [4] also estimated that the rate shift in diversification among angiosperms occurred at 213 Ma, following the divergence of Mesangiospermae which, following our age estimates, indicates a lag of 84–106 Myr. Ultimately, these results indicate that more precise age estimates require more precise hypotheses regarding the role of WGD in promoting evolutionary success. Given these long lag periods and that some, though clearly not all, clades that share a history of WGD are diverse or characterized by innovations, it requires more explicit hypotheses regarding which clades are considered successful.

Evidently, we find no direct support for the deterministic role of WGD in driving diversification or innovation. Rather, our data are more compatible with the more permissive model of evolution via genome duplication that emphasizes the importance of the post-WGD period of genome fractionation. During this period, the need to maintain a dosage balance of protein products selects for the maintenance of duplicates, followed by a relaxation of selection allowing sub- and neofunctionalization [7]. An additional consideration is the lineage specific re-diploidization model, which applies when species divergence occurs before the diploidization process in complete [44]. Under this model, the lag is produced by the pattern of tetrasomic inheritance that is characteristic of autopolyploidy, leading to massively delayed functional divergence of duplicate genes. This model also predicts that duplicate genes evolve independently in separate lineages, and that this can explain the divergent evolutionary trajectories of lineages that share the same history of WGD [44]. This more permissive model explains the ‘long fuse’ or ‘lag’ found in our results, whereby an early WGD during a lineage's evolution provides a primer for subsequent innovation and diversification, leading to the evolutionary success of both lineages [42]. It also explains the paucity of genes preserving all paralogues anticipated as a phylogenetic footprint of the *ζ* and *ɛ* WGD events, as a consequence of post-duplication dysploidy leading to dosage bias.

The quantification of this lag is clearly relevant to understanding the role of WGD in plant evolution [42]. Our methods are applicable to other WGD events characterized previously within the plant kingdom, including those thought to be associated with increased diversification or the K–Pg boundary [4,5]. Furthermore, these methods could be used to clarify the timing of the proposed WGDs associated with the origins and early evolution of vertebrates [45], which are still undermined by uncertainty around their timing.

## 5. Conclusion

Accurate and precise estimates of the timing of WGD events are fundamental to our understanding their significance on a macroevolutionary scale and can be achieved by coupling a careful appraisal of the fossil record with molecular clock approaches. We demonstrated that by concatenating multiple gene families with a shared history of WGD into a single alignment, the ages of two ancient WGD events, *ɛ* (angiosperm) and *ζ* (spermatophyte), were estimated to a high degree of precision. Both events were found to occur early in the stem of each lineage, predating the divergence of the crown groups by 50 Myr. These methods can be applied to date any previously characterized WGD event, including those identified in yeasts and vertebrates.

## Supplementary Material

Supplementary Figures

## Supplementary Material

Supplementary Tables

## Data accessibility

Electronic supplementary material includes Supplemental Experimental Procedures, three figures and three tables and can be found with this article online. The molecular sequence alignment and trees with fossil calibrations have been deposited in Figshare: https://figshare.com/s/d46377d5ae6999c0cd52.
