# Supplementary material for: Constraining the timing of whole genome duplication in plant evolutionary history
Source: Proc Biol Sci. 2017 Jul 5;284(1858):20170912. doi: 10.1098/rspb.2017.0912 (PMC5524505; doi:10.1098/rspb.2017.0912)
Supplement: Supplementary Figures [file rspb20170912supp1.docx]

##

## Supplementary Figure S1. An example of an orthogroup that was discarded from the analysis. Triple-Helix transcriptor family (ORTHO03D004565) was identified by Jiao *et al.* as containing the signal of the sigma duplication. Though not rejected, the signal is difficult to recover, due to an incongruent topology (paraphyletic gymnosperms) and the relationships of the two sets of paralogs not being clear.

##

##

## Supplementary Figure S2. The constrained tree used for molecular clock analyses. Constraints are show at each node where applied, with minimum constraints represent in blue, uniform constraints in red and the two duplication constraints in green. The equivalent halves of the duplication node have not been shown, although a matching set of constraints were cross-calibrated with them. Numbers correspond to the list of calibrations defined in Supplementary Table S2.
