# Supplementary material for: Constraining the timing of whole genome duplication in plant evolutionary history
Source: Proc Biol Sci. 2017 Jul 5;284(1858):20170912. doi: 10.1098/rspb.2017.0912 (PMC5524505; doi:10.1098/rspb.2017.0912)
Supplement: Supplementary Tables [file rspb20170912supp2.docx]

**Supplementary Information S1.** Full list of taxa included in the analyses and the source of the data

| Species | Order | Source | |
| --- | --- | --- | --- |
| **Mosses** |  | |  |
| *Sphagnum lescurii* | Sphagnales | | iPlant Data Store |
| *Physcomitrella patens* | Funariales | | Plaza 3.0 |
| *Ceratodon purpureas* | Dicranales | | iPlant Data Store |
| *Hedwigia ciliata* | Hedwigiales | | iPlant Data Store |
| *Thuidium delicatulum* | Hypnales | | iPlant Data Store |
| *Leucodon sciuroides* | Hypnales | | iPlant Data Store |
| *Anomodon attenuates* | Hypnales | | iPlant Data Store |
| *Rhynchostegium serrulatum* | Hypnales | | iPlant Data Store |
| *Bryum argenteum* | Bryales | | iPlant Data Store |
| *Rosulabryum capillare* | Bryales | | iPlant Data Store |
|  |  | |  |
| **Hornworts** |  | |  |
|  |  | |  |
| *Nothoceros aegnimaticus* | Dendroceratales | | iPlant Data Store |
| *Nothoceros vincentianus* | Dendroceratales | | iPlant Data Store |
|  |  | |  |
| **Liverworts** |  | |  |
| *Marchantia polymorpha* | Marchantiales | | iPlant Data Store |
| *Marchantia emarginata* | Marchantiales | | iPlant Data Store |
| *Ricciocarpos natans* | Marchantiales | | iPlant Data Store |
| *Sphaerocarpos texanus* | Sphaerocarpales | | iPlant Data Store |
| *Bazzania trilobata* | Jungermanniales | | iPlant Data Store |
| *Metzgeria crassipilis* | Metzgeriales | | iPlant Data Store |
|  |  | |  |
| **Lycophytes** |  | |  |
| *Selaginella moellendorfii* | Selaginellales | | Plaza 3.0 |
| *Selaginella stauntoniana* | Selaginellales | | iPlant Data Store |
| *Huperzia squarrosa* | Lycopodiales | | iPlant Data Store |
| *Pseudolycopodiella caroliana* | Lycopodiales | | iPlant Data Store |
| *Dendrolycopodium obscurum* | Lycopodiales | | iPlant Data Store |
|  |  | |  |
| **Ferns** |  | |  |
| *Equisetum diffusum* | Equisetales | | iPlant Data Store |
| *Psilotum nudum* | Psilotales | | iPlant Data Store |
| *Ophioglossum petiolatum* | Ophioglossales | | iPlant Data Store |
| *Angiopteris evecta* | Marattiales | | iPlant Data Store |
| *Alsophila spinulosa* | Cyatheales | | iPlant Data Store |
| *Pteridium aquilinum* | Polypodiales | | iPlant Data Store |
|  |  | |  |
| **Gymnosperms** |  | |  |
| *Picea abies* | Pinales | | GreenPhyl 4.0 |
| *Pinus taeda* | Pinales | | iPlant Data Store |
| *Cedrus libani* | Pinales | | iPlant Data Store |
| *Prumnopitus andina* | Pinales | | iPlant Data Store |
| *Cunninghamia lanceolata* | Pinales | | iPlant Data Store |
| *Juniperus scopulorum* | Pinales | | iPlant Data Store |
| *Taxus baccata* | Pinales | | iPlant Data Store |
| *Sciadopitys verticillata* | Pinales | | iPlant Data Store |
| *Zamia vasquezii* | Cycadales | | iPlant Data Store |
| *Cycas mycolitzii* | Cycadales | | iPlant Data Store |
| *Ginkgo biloba* | Ginkgoales | | iPlant Data Store |
| *Ephedra sinica* | Gnetales | | iPlant Data Store |
| *Gnetum montanum* | Gnetales | | iPlant Data Store |
| *Welwitschia mirabilis* | Gnetales | | iPlant Data Store |
|  |  | |  |
| **Angiosperms** |  | |  |
| *Amborella trichopoda* | Amborellales | | Plaza 3.0 |
| *Nuphar advena* | Nymphaeales | | iPlant Data Store |
| *Kadsura heteroclite* | Austrobaileyales | | iPlant Data Store |
| *Houttuynia cordata* | Piperales | | iPlant Data Store |
| *Saruma henryi* | Piperales | | iPlant Data Store |
| *Liriodendron tulipfera* | Magnoliales | | iPlant Data Store |
| *Persea americana* | Laurales | | iPlant Data Store |
| *Sarcandra glabra* | Chloranthales | | iPlant Data Store |
| *Acorus americanus* | Acorales | | iPlant Data Store |
| *Dioscorea villosa* | Dioscoreales | | iPlant Data Store |
| *Smilax bona-nox* | Liliales | | iPlant Data Store |
| *Colchicum autumnale* | Liliales | | iPlant Data Store |
| *Yucca filamentosa* | Asparagales | | iPlant Data Store |
| *Sabal bermudana* | Arecales | | iPlant Data Store |
| *Elaeis guineensis* | Arecales | | GreenPhyl 4.0 |
| *Phoenix dactylifera* | Arecales | | GreenPhyl 4.0 |
| *Musa acuminata* | Zingiberales | | Plaza 3.0 |
| *Musa balbisiana* | Zingiberales | | Phytozome |
| *Oryza sativa* | Poales | | Plaza 3.0 |
| *Panicum hallii* | Poales | | Phytozome |
| *Hordeum vulgare* | Poales | | Plaza 3.0 |
| *Sorghum bicolor* | Poales | | Plaza 3.0 |
| *Seteria italica* | Poales | | Plaza 3.0 |
| *Zea mays* | Poales | | Plaza 3.0 |
| *Brachypodium distachyon* | Poales | | Plaza 3.0 |
| *Escholzia californicum* | Ranunculales | | iPlant Data Store |
| *Aquiliegia formosa* | Ranunculales | | iPlant Data Store |
| *Podophyllum peltatum* | Ranunculales | | iPlant Data Store |
| *Beta vulgaris* | Caryophyllales | | Plaza 3.0 |
| *Diospyros malabarica* | Ericales | | iPlant Data Store |
| *Inula helenium* | Asterales | | iPlant Data Store |
| *Tanacetum parthenium* | Asterales | | iPlant Data Store |
| *Ipomoea purpurea* | Solanales | | iPlant Data Store |
| *Solanum tuberosum* | Solanales | | Plaza 3.0 |
| *Solanum lycospersicum* | Solanales | | Plaza 3.0 |
| *Rosmarinus officinales* | Lamiales | | iPlant Data Store |
| *Mimulus guttatus* | Lamiales | | Phytozome |
| *Catharanthus roseus* | Gentianales | | iPlant Data Store |
| *Coffea canephora* | Gentianales | | GreenPhyl 4.0 |
| *Allamanda cathartica* | Gentianales | | iPlant Data Store |
| *Vitis vinifera* | Vitales | | Plaza 3.0 |
| *Eucalyptus grandis* | Myrtales | | Plaza 3.0 |
| *Citrus sinensis* | Sapindales | | Plaza 3.0 |
| *Gossypium raimondii* | Malvales | | Plaza 3.0 |
| *Hibiscus cannabinus* | Malvales | | iPlant Data Store |
| *Theobroma cacao* | Malvales | | Plaza 3.0 |
| *Carica papaya* | Brassicales | | Plaza 3.0 |
| *Arabidopsis thaliana* | Brassicales | | Plaza 3.0 |
| *Arabidopsis lyrata* | Brassicales | | Plaza 3.0 |
| *Capsella rubella* | Brassicales | | Plaza 3.0 |
| *Capsella grandiflora* | Brassicales | | Phytozome |
| *Brassica rapa* | Brassicales | | Plaza 3.0 |
| *Thelungiella parvula* | Brassicales | | Plaza 3.0 |
| *Eutrema salsugineum* | Brassicales | | Phytozome |
| *Boechera stricta* | Brassicales | | Phytozome |
| *Linum usitatissimum* | Malpighiales | | Phytozome |
| *Populus trichocarpa* | Malpighiales | | Plaza 3.0 |
| *Ricinus communis* | Malpighiales | | Plaza 3.0 |
| *Manihot esculenta* | Malpighiales | | Plaza 3.0 |
| *Cucumis melo* | Cucurbitales | | Plaza 3.0 |
| *Cucumis sativus* | Cucurbitales | | Plaza 3.0 |
| *Citrullus lanatus* | Cucurbitales | | Plaza 3.0 |
| *Larrea tridentata* | Rosales | | iPlant Data Store |
| *Fragaria vesca* | Rosales | | Plaza 3.0 |
| *Prunus persica* | Rosales | | Plaza 3.0 |
| *Malus domestica* | Rosales | | Plaza 3.0 |
| *Boehmeria nivea* | Rosales | | iPlant Data Store |
| *Lotus japonicus* | Fabales | | Plaza 3.0 |
| *Cicer arietinum* | Fabales | | GreenPhyl 4.0 |
| *Cajanus cajan* | Fabales | | GreenPhyl 4.0 |
| *Glycine max* | Fabales | | Plaza 3.0 |
| *Medicago truncatula* | Fabales | | Plaza 3.0 |
|  |  | |  |
|  |  | |  |
|  |  | |  |
|  |  | |  |
|  |  | |  |
|  |  | |  |
|  |  | |  |
|  |  | |  |
|  |  | |  |
|  |  | |  |
|  |  | |  |
|  |  | |  |

**Supplementary Information S2 –** A full list of fossil calibrations updated for use in molecular clock analysis. All seed plants calibrations were applied twice across the tree, and each angiosperm calibration applied four times. Ages in millions of years before present.

**1. CG Embryophytes | MRCA: *Marchantia – Capsella* | 448.5 – 509 Ma**

**Fossil taxon and specimen.** Following Clarke *et al*.^1^, constraints were based on trilete spores from the Qusaiba-1 core from the Quasim formation of northern Saudi Arabia^2^ and Cambrian spores of the Bright Angel Shale in the lower elevations of the Grand Canyon, Arizona^3^

**Phylogenetic justification.** Following Clarke *et al.*^1^*,* the oldest records of liverworts date to the Early Devonian, however trilete spores support the total group Anthocerotae + Tracheophyta, providing a minimum constraint. The Cambrian spores of the Bright Angel Shale represent the oldest spores possessing two Embryophyte synapomorphies: permanent dyad and tetrad arrangements and multilamellate sporoderm*.*

**Minimum age.** 448.5 Ma.

**Maximum age.** 509 Ma.

**Age justification.** The minimum constraint, following Clarke et al.^1^, is based on the oldest occurrences of trilete spores, known from the Qusaiba-1 core from the Quasim Formation of northern Saudi Arabia. We follow Clarke *et al.*^1^ and accept a likely minimum age at the top of the *Acanthochitina barbata* biozone based on co-occurrence^3^, the base of which is estimated at 448.5 Ma., following Cooper *et al.*^4^. The maximum constraint is based on the Cambrian spores of the Bright Angel Shale, which falls fully within the span of the *Albertella, Glossopluera* and *Ehmaniella* trilobite biozones, representing 507.2-509 Ma.^5^

**2. CG Marchantiopsida | MRCA *Sphaerocarpos – Marchantia* | 228.4 Ma**

**Fossil taxon and specimen.** *Marchantites cyatheoides* [Plate 1A. number 13929 South African Museum Cape Town] from the Upper Umkomaas, Natal, Molteno Formation

**Phylogenetic justification.** Orginially assigned to the broad genus *Hepaticites* by Townrow^6^, however Anderson^7^ revised the taxon and placed it within the genus *Marchantites* based on the presence of a prostate forked thallus, a conspicuous midrib, rhizoides, air chambers and central scales, all indicating an affinity with the Marchantiaceae

**Minimum age.** 228.4 Ma.

**Age justification.** *Marchantites cyatheoides* is known only from the Molteno formation of South Africa and the Middle Triassic Sydney basin, Australia. The Molteno formation is among the most intensely studied Upper Triassic formations in the world, and based on the megaflora assemblages, was dated as Carnian by Anderson & Anderson^8^. As no formal boundary is defined for the Molteno formation, we took the upper boundary of the Carnian following Ogg^9^ as 228.4 Ma.

**3. SG Metzgeriales | MRCA *Bazzania – Metzgeria* | 407.6 Ma.]**

**Fossil taxon and specimen.** *Riccardiothallus devonicus* [CBY_n_9004008 Museum of Plant History, Institute of Botany, Chinese Academy of Sciences] from the Posongchong formation, Zhichang Village, Gumu Town, Wenshan District, Yunnan Province, China.

**Phylogenetic justification.** Guo et al.^10^ determined that *Riccardiothallus* shares several similarities with the extant genus *Riccardia* (Aneuraceae), including a flattened thallus with irregular branching, lack of conducting tissue and a lack of a costa, yet based on the age of the fossil, it was deemed most appropriate to assign it to a new genus.

**Minimum age. 407.6 Ma.**

**Age justification.** *Riccardiothallus* comes from the Posongchong formation in China, the stratigraphy of which was confirmed by Hao et al.^11^ as Lower Devonian (Pragian), based on the evidence of marine invertebrates from the overlying Pojiao formation. The upper limit of the Pragian (407.6 Ma.) was adopted as the minimum age following Becker *et al*.^12^

**4. CG Stomatophyta | MRCA: *Sphagnum* – Tracheophyta + Anthocerophyta | 426.7 – 509 Ma**

**Fossil taxon and specimen**. Following Clarke *et al****.*^1^** *Cooksonia cambrensis* [TCD22951, Department of Geology, Trinity College, Dublin] from the Devilsbit Mountain Area, Central Ireland was accepted as the oldest representative of total group Tracheophyta

**Phylogenetic justification.** The fossil record of mosses is poor and *Sporogonites* remains the oldest possible moss, though its phylogenetic position is too equivocal to provide a minimum constraint and so following Clarke *et al****.*^1^** *Cooksonia* was used to provide a minimum constraint, having been reinterpreted as a member of total group Tracheophyta rather than crown group^1^, on the basis that many of the characters that placed *Cooksonia* in the crown group are found only in younger specimens, and some of the characters, such as the presence of the sterome, are unlikely to be synapomorphies of the crown group^13^. Placing *Cooksonia* in the total group is congruent with unequivocal total group synapomorphies, such as multiple sporangia and differentially thickened tracheids^13^.

**Minimum age.** 426.7 Ma.

**Maximum age.** 509 Ma.

**Age justification.** Following Clarke *et al****.*^1^** the earliest occurrences of *Cooksonia* are bracketed by graptolites that are characteristic of the *ludensis* biozone, which coincides with the Wenlock-Ludlow series boundary^14^, providing a minimum age of 426.7 Ma. updated following Melchin *et al*.^15^. Also following Clarke *et al****.*^1^** the oldest members of total group Tracheophyta would likely have shared the poor fossilization characteristics as Bryophyte grade material, and is likely a poor approximation of the age of the clade, and so we followed a soft maximum age of 509 Ma.

**5. SG Bryidae | MRCA: *Thuidium – Bryum* | 259.7 Ma.**

**Fossil taxon and specimen.** *Campimirinus riopratense* [UNICAMP: CP1/155-195 at the University of Campinas] Teresina Formation (Permian–Guadalupian) collected in the Rio Preto Quarry in the state of Paraná, southern Brazil.

**Phylogenetic justification.** Though likened to the modern genus *Hypnum,* De Souza et al.^16^ were reluctant to assign *C. riopratense* to an extant clade based on the absence of double short costae in the gametophyte and other key diagnostic features and so favoured the creation of a new genus. Following Laenen et al.^17^ it was assigned to the Hypnales based on the similarity to early divergent pleurocarpous mosses.

**Minimum age.** 259.8

**Age justification.** Following De Souza *et al*.^16^, the Teresina Formation falls within the Passa Dois Group. Based on U/Pb isotopes, Santos *et al.*^18^ established the base age of this group as 270.6 +/- 0.7 Ma. As no formal upper boundary for the Terasina formation is established, a minimum age was constructed based on the upper boundary of the Guadalupian at 260.4 +/- 0.7 following Davydov *et al.*^19^

**6. MRCA: *Nothoceros – Huperzia* | 426.7 – 509 Ma.**

**Fossil taxon and specimen**. Following Clarke *et al****.*^1^***,* *Cooksonia cambrensis* [TCD22951, Department of Geology, Trinity College, Dublin] from the Devilsbit Mountain Area, Central Ireland was accepted as the oldest representative of total group Tracheophyta

**Phylogenetic justification.** Following Clarke *et al****.*^1^**, *Cooksonia* was reinterpreted as a member of total group Tracheophyta rather than crown group, on the basis that many of the characteris that placed *Cooksonia* in the crown group are found only in younger specimens, and some of the characters, such as the presence of the sterome, are unlikely to be synapomorphies of the crown group^13^. Placing *Cooksonia* in the total group is congruent with unequivocal total group synapomorphies, such as multiple sporangia and differentially thickened tracheids^13^.

**Minimum age. 426.7 Ma.**

**Soft maximum age. 509 Ma.**

**Age justification.** Following Clarke *et al****.*^1^** the earliest occurrences of *Cooksonia* are bracketed by graptolites that are characteristic of the *ludensis* biozone, which coincides with the Wenlock-Ludlow series boundary^14^, providing a minimum age of 426.7 Ma. updated following Melchin *et al*.^15^. Also following Clarke *et al****.*^1^** the oldest members of total group Tracheophyta would likely have shared the poor fossilization characteristics as Bryophyte grade material, and is likely a poor approximation of the age of the clade, and so we followed a soft maximum age of 509 Ma.

**7. CG Tracheophyta | MRCA: Lycophyta-Euphyllophyta | 422 Ma – 449.6 Ma.**

**Fossil taxon and specimen**. Clarke *et al*.^1^ based their calibration of this node on *Zosterophyllum* sp. [US384-8137; University of Saskatchewan Collections, Canada] from Bathurst Island^20^.

**Phylogenetic justification.** Following Clarke *et al****.*^1^** the *Zosterophyllum* sp. from Bathurst Island (Kotyk *et al*.^20^) is unequivocally zostrophyll given its possession of reniform sporangia, sporangia that dehisce along their distal margins, and laterally inserted sporangia. All *Zosterophyllum* species are total group Lycopsida^13^.

**Minimum age**. 422 Ma.

**Soft maximum age**. 449.5 Ma.

**Age justification**. *Zosterophyllum* sp. on Bathurst Island^20^ co-occurs with conodont *Ozarkodina douroensis*, which is restricted to the Ludlow (as O. n. sp. B in^4-7^. Thus, a minimum age interpretation can be derived from the top of the Ludlow, dated to 423.0 Ma ± 1.0 Myr, thus 422.0 Ma. The soft maximum constraint, following Clarke et al.^1^, is based on the oldest occurrences of trilete spores, known from the Qusaiba-1 core from the Quasim Formation of northern Saudi Arabia. We follow Clarke *et al.*^1^ and accept a likely a softa maximum at the top of the *Acanthochitina barbata* biozone based on co-occurrence^3^, the base of which is estimated at 449.5 Ma, following Cooper *et al.*^4^.

**8. CG Lycophytes | MRCA: *Huperzia-Selaginella* | 392.1 Ma – 449.5 Ma.**

**Fossil taxon and specimen.** *Leclercquia complexa* [CW092 (07 – 061): Collections of the Centre for Palynological Studies, Department of Animal and Plant Sciences, University of Sheffield, UK], from Campbellton Formation outcropping on the south shore of the Restigouche River, between Dalhousie and Campbellton, New Brunswick, eastern Canada^21^.

**Phylogenetic justification.** Kenrick and Crane^3^ identified *Leclercquia complexa* as the oldest member of Isoetopsida and crown Lycopodiophyta. This interpretation is supported by spore characteristics analysed phylogenetically by Wellman et al.^21^.

**Minimum age.** 392.1 Ma.

**Soft Maximum age.** 449.5 Ma.

**Age justification.** A Late Emsian age is often cited for the New Brunswick occurrences of identified *Leclercquia complexa* e.g. ^22^ and, indeed, the *Stockmensella-Leclerqia* macroplant Biozone spans all but the earliest Emsian^12^. However, Wellman et al.^22^ attribute their own material of *Leclercquia complexa* to the middle of the *Emphanisporites annulatus – Camarozonotriletes sextantii* Spore Assemblage Biozone which falls within the early part of the Emsian. In either instance, the earliest records of *Leclercquia complexa* fall fully within the Emsian, the end of which is dated to 393.3 Ma ± 1.2 Myr^12^, yielding a minimum constraint of 392.1 Ma. The soft maximum constraint, following Clarke et al.^1^, is based on the oldest occurrences of trilete spores, known from the Qusaiba-1 core from the Quasim Formation of northern Saudi Arabia. We follow Clarke *et al.*^1^ and accept a likely a softa maximum at the top of the *Acanthochitina barbata* biozone based on co-occurrence^3^, the base of which is estimated at 449.5 Ma., following Cooper *et al.*^4^.

**Discussion.** Magallon et al.^23^ cite a minimum age of 385 Ma, based on the Middle-Upper Devonian Boundary, but our more detailed stratigraphy allows for an older minimum age interpretation of *Leclercquia complexa.*

**9. CG Euphyllophytes | MRCA: Monilophyta-Spermatophyta | 385.571 Ma – 449.5 Ma.**

**Fossil taxon and specimen**. *Rellimia thomsonii* from the Panther Mountain Formation of New York^24^ [335.34; Paleobotanical Collection of the State University of New York at Bingham].

**Phylogenetic justification.** Magallón et al.^23^ identified *Ibyka amphikoma*^25^ as the oldest record of the pteridophyte lineage based on phylogenetic analyses undertaken by Kenrick and Crane^13^.

**Minimum age**. 384.71 Ma.

**Soft maximum age**. 449.5 Ma.

**Age justification**. Clarke et al.^1^ proposed *Rellimia thomsonii*, an aneurophytalean progymnosperm from the Panther Mountain Formation of New York^24^, as the oldest record of crown Euphyllophyta. The Panther Mountain Formation is equivalent to the Ludlowville and Skaneateles formations^1^, which occur below the Moscow Formation of New York^26^, making *Rellimia thomsonii* older than *Ibyka amphikoma*^1^*.* The Ludlowville-Moscow formation boundary falls deep within the Lower *varcus* zone^27^ and, therefore, below the *rhenanus-ansatus* biozonal boundary^12^, at the very least, which has been dated to 386.25 Ma ± 0.679 Myr, yielding a minimum constraint of 385.571 Ma. The soft maximum constraint, following Clarke et al.^1^, is based on the oldest occurrences of trilete spores, known from the Qusaiba-1 core from the Quasim Formation of northern Saudi Arabia. We follow Clarke *et al.*^1^ and accept a likely a softa maximum at the top of the *Acanthochitina barbata* biozone based on co-occurrence^3^, the base of which is estimated at 449.5 Ma., following Cooper *et al.*^4^.

**Discussion.** Magallón et al.^23^ established a minimum age constraint using *Ibyka amphikoma,* based on the Givetian-Frasnian boundary, for which they provided a date of 385 Ma, though this has since been revised to 382.7 Ma ± 1 Myr^12^. *Ibyka amphikoma* was recovered from the Manorkill Shale Member, which is a lateral equivalent of the Windom Member, within the Moscow Formation of New York^28,29^, which falls fully within the *ansatus* conodont Biozone^30,31^ the top of which is dated to 385.41 Ma ± 0.7 Myr^12^, thus, yielding a minimum age constraint of 384.71 Ma, younger than the minimum age of *Rellimia thomsonii*.

**10. CG Monilophytes | MRCA: *Equisetum - Pteridium* | 384.71 Ma – 449.5 Ma.**

**Fossil taxon and specimen.** *Ibyka amphikoma* was recovered from the Manorkill Shale Member at Schoharie Creek directly below the spillway of Gilboa dam, Gilboa, Schoharie County, New York, Gilboa^25^.

**Phylogenetic justification.** *Ibyka amphikoma*^25^ is the oldest record of the equisetopsid lineage based on the phylogenetic analyses undertaken by Kenrick and Crane^13^.

**Minimum age.** 384.71 Ma.

**Soft Maximum age.** 449.5 Ma.

**Age justification.** *Ibyka amphikoma* was recovered from the Manorkill Shale Member, which is a lateral equivalent of the Windom Member, within the Moscow Formation of New York^28,29^, which falls fully within the *ansatus* conodont Biozone^30,31^ the top of which is dated to 385.41 Ma ± 0.7 Myr ^12^, thus, yielding a minimum age constraint of 384.71 Ma. The soft maximum constraint, following Clarke et al.^1^, is based on the oldest occurrences of trilete spores, known from the Qusaiba-1 core from the Quasim Formation of northern Saudi Arabia. We follow Clarke *et al.*^1^ and accept a likely a softa maximum at the top of the *Acanthochitina barbata* biozone based on co-occurrence^3^, the base of which is estimated at 449.5 Ma., following Cooper *et al.*^4^.

**Discussion.** Magallón et al.^23^ established a minimum age constraint based on *Ibyka amphikoma* using the Givetian-Frasnian boundary, for which they provided a date of 385 Ma, though this has since been revised to 382.7 Ma ± 1 Myr^12^. However, we provide a more detailed stratigraphic justification for the age of *I. amphikoma* which allows for an older minimum age constraint.

**11. SG Leptosporangiate ferns | MRCA: *Angiopteris – Pteridium |* 315.1 Ma*.***

**Fossil taxon and specimen.** *Senftenbergia plumosa* [E3672, National Museum, Prague] from the Kladno formation of the Nyrany locality in the Pilsen Basin, Bohemian Massif^32^

**Phylogenetic justification.** Despite similar reproductive tissues to members of the Schizeaceae, *Senftenbergia plumosa* assigned to the Tedeleaceae based on angular diametric cells following Pšenička and Bek^32^ following careful examination of the epidermal cells and cuticular layer.

**Minimum age.** 315.1 Ma.

**Age justification.** *S. plumosa* occurs throughout the Westphalian A to the Lower Permian following Bek and Pšenička^33^, and so the upper limit of the Westphalian A was accepted as a minimum constraint. Unfortunately, the boundary of the Westphalian A does not correlate with the current Geologic Time Scale, and so the upper boundary of the Westphalian B (315.2 +/- 0.1) was taken as the minimum age following Davydov et al.^19^

**12. SG Polypodiales | MRCA: *Alsophila – Pteridium |* 98.79 Ma.**

**Fossil taxon and specimen.** *Krameropteris resinatus* [AMNH Bu-ASJH-3] from Amber mines near Tanai in Kachin State, Myanmar^34^.

**Phylogenetic justification.** Schmidt et al.^34^ assigned *K. resinatus* to the Dennstaedtiaceae based on the presence of polypod sporangia, free-veined leaves and exindusiate sori. However, irregular tuber shaped structures on the leaves are unique among extant ferns and so it was assigned to its own genus^34^.

**Minimum age.** 98.79 Ma.

**Age justification.** Biostratigraphic studies suggested a late Albian age of the amber-bearing sediment (Cruickshank and Ko^35^) hence the inclusions have a late Early Cretaceous age, with a minimum age of

98.79 million years (earliest Cenomanian, early Late Cretaceous) that is based on recent U-Pb dating of zircons (Shi et al.^36^).

**13. CG Spermatophytes | MRCA: *Ginkgo-Capsella*| 308.14 Ma – 365.629 Ma.**

**Fossil taxon and specimen**. *Cordaites iowensis* [UIC 12,233: University of Illinois at Chicago; OUPH 9616- 9742: Ohio University Paleobotanical Herbarium, Department of Botany, Ohio University, Athens, Ohio] from the Laddsdale Coals (Cherokee Group, Desmoinesian) near What Cheer, Iowa^37^.

**Phylogenetic justification.** Clarke *et al*.^1^ identify cordaitean coniferophytes as the oldest records of the crown group of the spermatophyte clade. The oldest whole plant reconstruction is *Cordaites iowensis* from the Laddsdale Coals (Cherokee Group, Desmoinesian) near What Cheer, Iowa^37^.

**Minimum age**. 308.14 Ma.

**Soft maximum age**. 365.629 Ma.

**Age justification**. Janousek and Pope^38^ argue that the Laddsdale Coal is equivalent to the Bluejacket Coal of Oklahoma, which occurs as part of the Bluejacket Sandstone Member, underlying the Inola Limestone, part of the Inola Cyclothem of the Krebs subgroup of the Cherokee Group, characterized by the occurrence of the conodonts *Idiognathodus amplificus, Idiognathodus podolskensis and Neognathodus asymmetricus*^39^. The Inola cyclothem falls fully within the *Idiognathodus amplificus/ Idiognathodus obliquus* biozone^40^. This is indicative of the *Neognathodus medexultimus*-*Streptognathodus concinnus* (Pc10) biozone, certainly older than the *Neognathodus roundyi* – *Streptognathodus cancellosus* (Pc11) biozone^19,40^. The base of Pc10 is bracketed by an older age constraint of 312.01 Ma ± 0.37 Myr and the base of Pc11 is bracketed by a younger age constraint of 308.5 Ma ± 0.36 Myr in the Composite Standard of Davydov et al.^19^, yielding a minimum constraint of 308.14 Ma.

The soft maximum constraint follows Clarke et al.^1^ who based theirs on the first records of seeds in the form of preovules that satisfy the criteria of the seed habit, which occur in the Upper Fammenian (Late Devonian) VCo Spore Biozone^41^, a well documented example of which being *Elkinsia polymorpha*^42^; *E. polymorpha* has been recovered from the Hampshire Formation, West Virginia, from which the palynomorphs *Grandispora cornuta, Retispora macroreticulata, Retusotriletes phillipsii* and *Rugospora radiata* have been reported^43^, which substantiate assignment to the VCo Biozone^44^. The VCo biozone is not directly dated but its base falls within the *Palmatolepis trachytera* conodont biozone^45^, the base of which is dated to 364.19 Ma ± 1.439 Myr^12^, yielding a soft maximum constraint on the divergence of crown Spermatophyta at 365.629 Ma.

**14. CG Acrogymnosperms | MRCA: *Ginkgo-Pinus* | 308.14 Ma – 365.629 Ma.**

**Fossil taxon and specimen.** *Cordaites iowensis* [UM4616: University of Michigan and Illinois Geological Survey, Ann Arbor MI, USA] from the Laddsdale Coals (Cherokee Group, Desmoinesian) near What Cheer, Iowa, USA^37^.

**Phylogenetic justification.** Clarke *et al*.^1^ identify cordaitean coniferophytes as the oldest records of the *Ginkgo-Pinus* clade, the oldest whole plant reconstruction of which is *Cordaites iowensis* from the Laddsdale Coals (Cherokee Group, Desmoinesian) near What Cheer, Iowa^37^.

**Minimum age.** 308.14 Ma.

**Soft Maximum age:** 365.629 Ma.

**Age justification.** Janousek and Pope^38^ argue that the Laddsdale Coal is equivalent to the Bluejacket Coal of Oklahoma, which occurs as part of the Bluejacket Sandstone Member, underlying the Inola Limestone, part of the Inola Cyclothem of the Krebs subgroup of the Cherokee Group, characterized by the occurrence of the conodonts *Idiognathodus amplificus, Idiognathodus podolskensis and Neognathodus asymmetricus*^39^. The Inola cyclothem falls fully within the *Idiognathodus amplificus/ Idiognathodus obliquus* biozone^40^. This is indicative of the *Neognathodus medexultimus*-*Streptognathodus concinnus* (Pc10) biozone, certainly older than the *Neognathodus roundyi* – *Streptognathodus cancellosus* (Pc11) biozone^19,40^. The base of Pc10 is bracketed by an older age constraint of 312.01 Ma ± 0.37 Myr and the base of Pc11 is bracketed by a younger age constraint of 308.5 Ma ± 0.36 Myr in the Composite Standard of Davydov et al.^19^, yielding a minimum age constraint of 308.14 Ma. A soft maximum is based upon the first appearance of seeds in the form of preovules which are attributable to the spermatophyte stem, the oldest interpretation of which is 365.629 Ma (see Spermatophyta).

**Discussion**. Zanne et al.^46^ derive a minimum constraint from *Emporia lockardii* at 290.0 Ma which they recognize as a member of crown-Acrogymnospermae within a phylogenetic concept of the group in which, as here, cycads and *Ginkgo* comprise a clade.

**15. MRCA: *Ginkgo-Cycas* | 264.7 Ma – 365.629 Ma.**

**Fossil taxon and specimen.** *Crossozamia chinensis* [GP0027: Beijing Graduate School, China Institute of

Mining, Beijing, China], Lower Shihhotse Formation at Simugedong, Dongshan (East Hills), Taiyuan, north China^47^.

**Phylogenetic justification.** Nagalingum et al.^48^ identify *Crossozamia* as the oldest record of the *Cycas* lineage, based on megasporophylls that exhibit similarity to extant *Cycas*^49^. They argue against the interpretation of *Crossozamia* as the sister lineage of *Cycas* based on the presence of an estipulate leaf base and a terminal pinna found in the seedlings^49^, instead favouring its assignment to the cycad stem. The arguments presented clearly raise doubts about the assignment of *Crossozamia* to crown-cycads, however, they do not provide definitive evidence of its exclusion from this clade and so *Crossozamia* may more appropriately be assigned to the cycad total group (i.e. we cannot discriminate between a stem or crown-cycad affinity based on the available evidence). In either instance, *Crossozamia* is the oldest record of the minimal clade comprised of *Gingko* and *Cycas*.

**Minimum age.** 264.7 Ma.

**Soft Maximum age.** 365.629 Ma.

**Age justification.** The Lower Shihhotse Formation at Simugedong, Dongshan (East Hills), Taiyuan, north China^47^ has been established biostratigraphically as Roadian-Wordian (middle Permian)^50^ and, thus a minimum age constraint can be established on the Wordian-Capitanian Boundary which has been dated to 265.1 Ma ± 0.4 Myr^51^. Thus, the minimum age constraint on the *Cycas-Ginkgo* clade is 264.7 Ma. A soft maximum is based upon the first appearance of seeds in the form of preovules which are attributable to the spermatophyte stem, the oldest interpretation of which is 365.629 Ma (see Spermatophyta).

**16. CG Conifers | MRCA: Pinus-Cunninghamia | 147 Ma - 312.38 Ma.**

**Fossil taxon and specimen.** *Araucaria mirabilis* [NHM V. 30953: Natural History Museum, London, UK], represented by cones, from Cerro Cuadrado petrified forest, La Matilde Formation, Patagonia, Argentina^52-55^.

**Phylogenetic justification.** These fossils possess a ‘vascular plexus’ at the ovule base, ovuliferous scale vascularization, two vascular strands to the conescale complex and an embryo with two cotyledons, all characters established to distinguish *Araucaria* section *Bunya* of the Araucariaceae^54,56^, to which only extant *Araucaria bidwillii* belongs.

**Minimum age.** 147 Ma.

**Soft Maximum age:** 312.38 Ma.

**Age justification.** The age of La Matilde Formation is poorly constrained as the stratigraphy is complex, although the volcanic deposits do allow radiometric dating. La Matilde Formation is overlain by volcanics dated to 157 Ma ± 10 Myr^57^, and thus the minimum constraint on the divergence of crown Cupressophyta, total group Cupressophyta and crown Coniferae is 147 Ma. A soft maximum constraint can be based on *Cordaites iowensis*, a cordaitean coniferophyte from the Laddsdale Coals (Cherokee Group, Desmoinesian) near What Cheer, Iowa^37^, is the oldest whole plant reconstruction for Coniferae. Janousek and Pope^23^ argue that the Laddsdale Coal is equivalent to the Bluejacket Coal of Oklahoma, which occurs as part of the Bluejacket Sandstone Member, underlying the Inola Limestone, part of the Inola Cyclothem of the Krebs subgroup of the Cherokee Group, characterized by the occurrence of the conodonts *Idiognathodus amplificus, Idiognathodus podolskensis and Neognathodus asymmetricus*^24^. The Inola cyclothem falls fully within the *Idiognathodus amplificus/ Idiognathodus obliquus* biozone^40^. This is indicative of the *Neognathodus medexultimus*-*Streptognathodus concinnus* (Pc10) biozone, certainly older than the *Neognathodus roundyi* – *Streptognathodus cancellosus* (Pc11) biozone^19,40^. The base of Pc10 is bracketed by an older age constraint of 312.01 Ma ± 0.37 Myr and the base of Pc11 is bracketed by a younger age constraint of 308.5 Ma ± 0.36 Myr in the Composite Standard of Davydov et al.^19^, yielding a soft maximum of 312.38 Ma.

**Discussion.** This is the fundamental divergence of Coniferae into Cupressophyta, Gnetales and Pinaceae. The oldest secure records of the gnepine total group occur within the Yixian Formation of Liaoning, China, the minimum age of which is 121.8 Ma (see^1^). The oldest possible records of Cupressophyta total group include Triassic *Rissikia media* (Townrow, 1967) but it lacks the Podocarpaceae diagnostic feature of one ovule per cone scale, instead possessing two^1^. Other Triassic-Jurassic records are equally problematic^58-60^.

**17. CG Gnetales | MRCA: *Gnetum-Welwitschia* | 119.6 Ma – 312.38 Ma.**

**Fossil taxon and specimen.** *Eoantha zherikhinii* [Repository of the Institute of Biology and Pedology, Vladivostok, Russia], from the Zaza Formation at the Baisa locality in the upper reaches of the Vitim River in Lake Baikal^61^.

**Phylogenetic justification.**

**Minimum age.** 119.6 Ma.

**Soft Maximum age.** 312.38 Ma.

**Age justification.** The Zaza Formation can be correlated with the Turga Formation, also of Transbaikalia based principally on common elements of their floral assemblages, including *Asteropollis asteroids*, *Dicotylophyllum pusilum*, *Baisa hirsuita, Podozamites, Schizolepis, Pseudolarix, Phoenicopsis, Czekanowskia rigida* and *Sphenobaiera*^61-64^. The age of the Turga flora and Formation is based on the chronological distribution of *Asteropollis* type pollen, but correlation with the Yixian Formation of China is also supported strongly^62^, allowing for refinement of the *Asteropollis*-derived ages. Correlation between Turga and Yixian is based on similarities in the floral assemblages of these two formations, with the shared presence of the species *Baisa hirsuita, Botrychites reheensis, Neozamites verchojanensis, Pityolepis pseudotsugaoides, Brachyphyllum longispicum, Scarbugia hilii, Ephedrites chenii, Carpolithus multiseminalis,* *Carpolithus pachythelis, Schizolepis, Baiera, Coniopteris, Ginkoites, Pityocladus* , *Pityospermum* and *Elatocladus*^61,62,65^. The shared presence of *Asteropollis asteroides* in Turga and Zaza can be used to constrain their age. The last appearance of *Asteropollis* pollen is in Antarctica^66^ and is dated to the end-Campanian, at the latest 72.1 Ma ± 0.2^67^. This minimum may be constrained further based on the correlation of the Zaza Formation through the Turga Formation to the Yixian Formation. The main fossil bearing beds in the Yixian Formation have been recently dated and may be as old as 129.2 Ma^68^, however, in the absence of knowledge of the position of the fossils within the stratigraphy, relative to the sources of the absolute dates, a minimum age constraint can be derived from the Jiufontang Formation which overlies it. ^40^Ar/^39^Ar dating of a number of samples from the Jiufontang Formation has yielded an age of 120.3 ± 0.7 Ma for the volcanic tuffs^69^, establishing a minimum constraint of 119.6 Ma for the age of the Yixian, Formation and, thus ultimately the Zaza Formation.

A soft maximum constraint can be based on *Cordaites iowensis*, a cordaitean coniferophyte from the Laddsdale Coals (Cherokee Group, Desmoinesian) near What Cheer, Iowa^37^, is the oldest whole plant reconstruction for Coniferae. Janousek and Pope^38^ argue that the Laddsdale Coal is equivalent to the Bluejacket Coal of Oklahoma, which occurs as part of the Bluejacket Sandstone Member, underlying the Inola Limestone, part of the Inola Cyclothem of the Krebs subgroup of the Cherokee Group, characterized by the occurrence of the conodonts *Idiognathodus amplificus, Idiognathodus podolskensis and Neognathodus asymmetricus*^39^. The Inola cyclothem falls fully within the *Idiognathodus amplificus/ Idiognathodus obliquus* biozone^40^. This is indicative of the *Neognathodus medexultimus*-*Streptognathodus concinnus* (Pc10) biozone, certainly older than the *Neognathodus roundyi* – *Streptognathodus cancellosus* (Pc11) biozone^19,40^. The base of Pc10 is bracketed by an older age constraint of 312.01 Ma ± 0.37 Myr and the base of Pc11 is bracketed by a younger age constraint of 308.5 Ma ± 0.36 Myr in the Composite Standard of Davydov et al.^19^, yielding a soft maximum of 312.38 Ma.

**18. CG Angiosperms | MRCA: *Amborella-Austrobuxus* | 125.9 Ma – 247.3 Ma.**

**Fossil taxon and specimen**. Tricolpate pollen grain [BRN 126] from the Cowleaze Chine Member of the Vectis Formation of the Isle of Wight^70^.

**Phylogenetic justification.** Following Clarke at al.^1^, our minimum age constraint is based on the earliest occurrences Fischer’s rule tricolpate pollen, and knowledge of the distribution of tricoplate pollen across the phylogeny of angiosperms^71^.

**Minimum age**. 125.9 Ma.

**Soft maximum age**. 247.3 Ma.

**Age justification.** Following Clarke at al.^1^, the Cowleaze Chine Member of the Vectis Formation of the Isle of Wight^70^ occurs within the M1n polarity chron at the top of the Barremian, dated as 126.3 Ma ± 0.4 Myr^67^. The soft maximum age constraint is based on sediments devoid of angiosperm-like pollen below their first report in the Middle Triassic, thus, the base of the Anisian, dated to 247.1 Ma ± 0.2 Myr^35^, thus, 247.3 Ma.

**Discussion.** The recently described *Euanthus panii*^72^, *Juraherba bodae*^73^ and *Yuhania dahugouensis*^74^ from the Jiulongshan Formation were considered but not assigned. At the current stage, the age of the formation appears to be still not fully settled despite most experts agree on a middle Jurassic age (see^73,74^), whereas the assignment to extant lineages also required further investigation using phylogenetic approaches to confirm the proposed relationships of *Juraherba* to Hydatellaceae - which are the sister to the remaining Nymphaeales lineage and *Yuhania* to monocots.

**19. SG Nympheales | MRCA: *Nymphaea-Kadsura* | 110.87 Ma.**

**Fossil taxon and specimen.** *Pluricarpellatia* *peltata* [MB.Pb. 2000/80: Museum of Natural History, Berlin, Germany], from the Crato Formation of Brazil^75^

**Phylogenetic justification.** *Pluricarpellatia* *peltata* has been considered phylogenetically and resolved as members of the lineage leading to *Cabomba* after it diverged from *Nymphaea*^76^.

**Minimum age.** 110.87 Ma.

**Age justification.** Clarke et al.^1^ argued that the age of the Crato Formation could not be constrained to being definitively older than Albian based on pollen^77^, ostraco^78^ and dinoflagellate^79^ biostratigraphy and, in the absence of further evidence, established a minimum constraint on the Albian-Cenomanian boundary. Massoni et al.^80^ argued for an Aptian age for the Crato Formation based on evidence from Heimhofer and Hochuli^79^ but, unfortunately, these authors do not present evidence that can discriminate against a possible early Albian age for the Crato Formation, as acknowledged by Mohr et al.^81^. While the evidence suggests, at worst, an early Albian age for the Crato Formation, it is possible to derive a minimum age interpretation for the Formation based on the Early-Middle Albian Boundary, which coincides approximately with the base of the *Douvilleiceras mammillatum* ammonite biozone, dated to 110.87 Ma^67^.

**Discussion.** Magallon et al.^23^ derive a minimum constraint from *Monetianthus* *mirus* which they recognize as a representative of the Nymphaeaceae stem lineage and, thus, use it as the basis of a minimum constraint on the age of total-group Nymphaceaceae at 125 Ma. However, Clarke et al.^1^ demonstrated that the minimum age of the host deposit, Vale de Água, Portugal^82,83^ is 93.9 Ma^67^. However, there are other, potentially older records of Nymphaeaceae and, more specifically, the crown clade circumscribed by *Nymphaea-Cabomba*. Clarke et al.^1^ identified much older, but more equivocal records, as well as the oldest unequivocal records, viz. *Pluricarpellatia* *peltata* from the Crato Formation of Brazil^75^ and *Scutifolium jordanicum* from the Jarash Formation (Kurnub Group) of Jordan^76^, both of which have been considered phylogenetically and resolved as members of the lineage leading to *Cabomba* after it diverged from *Nymphaea*^76^. *Scutifolium jordanicum* was used to establish a minimum age for crown-Nymphaeales at 105 Ma by Smith et al.^84^, and for total-group Cabombaceae at 105 Ma by Zanne et al.^46^. The Jarash Formation can be dated minimally to 95 Ma (96.1 Ma ± 1.1 Myr in^85^, but the Crato Formation is older .

**20. SG Austrobaileyales | MRCA: *Kadsura* - *Capsella*| 107.59 Ma.**

**Fossil taxon and specimen.** *Anacostia* *virginiensis* [PP44151 ] from the Puddledock locality, Tarmac Lone Star Industries sand and gravel pit, Virginia USA^86^.

**Phylogenetic justification.**  Originally ascribed as an early magnoliid or monocot^86^, Doyle et al*.*^87^ resolved through phylogenetic analysis that *Anacostia* belongs within the Austrobaileyales based on the presence of several synapomorphies including a sclerotic mesotesta, palisade exotesta and basal ovule position.

**Minimum age.** 107.59 Ma.

**Age justification.** Massoni et al.^80^ reason that the sediments in the Puddledock Locality are definitively early Albian based on the presence of reticulate tricolpate pollen and *Clavatipollenites rotundus* (aff. *Retimonocolpites dividuus*^88^) but not striate tricolpates, which occur later in the early Albian. Therefore, they constrain minimally the age of the *A. virginiensis* by the Middle-late Albian boundary, which coincides with the base of the *Diploceras cristatum* biozone which has been dated to 107.59 Ma^67^.

**Discussion**. *Anacostia*, reportedly from the early and middle Albian of Buarcos, Famalicão, and Vale de Agua (Portugal), Puddledock (Virginia, USA), and Kenilworth (Maryland, USA) was recognized as the oldest fossil record of the Austrobaileyales^89,90^. Doyle and Endress^90^ *identified* *Anacostia* *portugallica* and *A. teixeirae* as early Albian and, therefore the oldest species belonging to this lineage. However, the minimum age interpretation of these localities the Figueira da Foz Formation cannot be constrained minimally to more than 92.8 Ma (see above). However, the minimum age constraint on *A. virginiensis* from the Puddledock Locality is older.

**21. CG Mesangiosperms | MRCA: *Sarcandra – Capsella* | 125.9 Ma – 247.3 Ma.**

**Fossil taxon and specimen**. Tricolpate pollen grain [BRN 126] from the Cowleaze Chine Member of the Vectis Formation of the Isle of Wight.^70^.

**Phylogenetic justification.** Following Clarke at al.^1^, our minimum age constraint is based on the earliest occurrences Fischer’s rule tricolpate pollen, and knowledge of the distribution of tricoplate pollen across the phylogeny of angiosperms^71^.

**Minimum age**. 125.9 Ma.

**Soft maximum age**. 247.3 Ma.

**Age justification.** Following Clarke at al.^1^, the Cowleaze Chine Member of the Vectis Formation of the Isle of Wight^70^ occurs within the M1n polarity chron at the top of the Barremian, dated as 126.3 Ma ± 0.4 Myr^67^. The soft maximum age constraint is based on sediments devoid of angiosperm-like pollen below their first report in the Middle Triassic, thus, the base of the Anisian, dated to 247.1 Ma ± 0.2 Myr^9^, thus, 247.3 Ma.

**22. CG Magnoliales | MRCA: *Liriodendron - Persea* | 110.87 Ma.**

**Fossil taxon and specimen.** *Endressinia brasiliana* [MB. PB. 2001/1455: Museum of Natural History, Institute of Paleontology, Berlin, Germany], from the Crato Formation of Brazil^90^.

**Phylogenetic justification.** Masson et al. identify both *Schenkeriphyllum glanduliferum* and *Endressinia brasiliana*, both from the Crato Formation of Brazil^90,91^, as the oldest records of crown Magnoliineae, the sister clade of Myristicaceae ^92^, based on the phylogenetic analyses^89,90,91^.

**Minimum age.** 110.87 Ma.

**Age justification.** Clarke et al.^1^ argued that the age of the Crato Formation could not be constrained to being definitively older than Albian based on pollen^77^, ostracod^78^, and dinoflagellate^79^ biostratigraphy and, in the absence of further evidence, established a minimum constraint on the Albian-Cenomanian boundary. Massoni et al.^80^ argued for an Aptian age for the Crato Formation based on evidence from Heimhofer and Hochuli^79^ but, unfortunately, these authors do not present evidence that can discriminate against a possible early Albian age for the Crato Formation, as acknowledged by Mohr et al.^92^. While the evidence suggests at worst, an early Albian age for the Crato Formation, and so it is possible to derive a minimum age interpretation for the Formation based on the Early-Middle Albian Boundary, which coincides approximately with the base of the *Douvilleiceras mammillatum* ammonite biozone, dated to 110.87 Ma^67^.

**Discussion.** *Archaeanthus linnenbergii* was recognized as a further putative stem group Magnoliaceae but it is younger than *Endressinia*^80,89^.

**23. SG Saururaceae| MRCA: *Saruma-Houttuynia* | 44.3 Ma.**

**Fossil taxon and specimen.** *Saururus tuckerae* [UAPC P1631 Bbot a: University of Alberta (Edmonton) Paleobotanical Collections] from the Middle Eocene Princeton Chert, British Columbia, Canada.

**Phylogenetic justification.** Massoni et al.^80^ follow Smith and Stockey^94^ in identifying *Saururus tuckerae* as the oldest record of total group *Saururus*. Based on tens of flowers and a partial inflorescence, the flower structure and pollen are characteristic of Saururaceae (Piperales), and phylogenetic analyses resolved *S. tuckerae* as the sister clade to extant *Saururus*^94^.

**Minimum age.** 44.3 Ma.

**Age justification.** The Princeton Chert is part of the Allenby Formation which has been the subject of a number of absolute dating studies yielding age estimates of 48 Ma ± 2 Myr^95,96^, between 47 Ma ± 2 Myr and 50 Ma ± 2 Myr^97^, between 46.2 Ma ± 1.9 Myr and 49.4 Ma ± 2 Myr^98^, and 52.08 Ma ± 0.12 Myr^99^ for the Allenby Formation. We follow Massoni et al.^80^ in basing our minimum constraint based on the youngest age Interpretation of the youngest radiometric age estimate, viz. 44.3 Ma

**24. CG Monocots | MRCA: *Acorus-Oryza* | 112.6 Ma.**

**Fossil taxon.** The earliest records of *Liliacidites* occur at the Trent’s Reach Locality of the Potomac Group, attributable to the Albian Zone I^100^.

**Phylogenetic justification.** Doyle et al.^87^ identified pollen referred to the genus *Liliacidites* (but not Similipollis) as represesentative of the monocot stem, making it the oldest secure record of the monocot total group (see^89^).

**Minimum age.** 112.6 Ma.

**Age justification.** In the absence of further stratigraphic constraint, these earliest records of *Liliacidites* can be constrained in age by the Aptian-Albian Boundary, dated to 113.0 ma ± 0.4 Myr, thus, 112.6 Ma.

**Discussion.** Doyle et al.^87^ highlight that, despite decades of sampling of the Hauterivian and Barremian of England, no clear representatives of *Liliacidites* pollen have been recovered^101^, perhaps implying that the earliest records from the Albian are a close approximation of their antiquity. Because of the position of monocots in our molecular tree we consider *Liliacidites* to be nested within monocots, and use it to calibrate the monocot crown node.

**25. CG Coryphoideae | MRCA: *Sabal-Oryza* | 83.41 Ma.**

**Fossil taxon and specimen.** *Sabalites carolinensis* [PAL 175717/P 38208: Smithsonian Museum of Natural History; Washington DC, USA] described from the Middendorf Arkose Member of Black Creek Formation near Langley, Aiken County, South Carolina^102^.

**Phylogenetic justification.** The phylogenetic relationships of this fossil have been discussed in Hertweck et al.^103^ and Iles et al.^104^.

**Minimum age.** 83.41 Ma

**Age justification.** Berry’s view that the Middendorf was merely a distinct facies within the Black Creek Formation, rather than a stratigraphically distinct unit, has been rejected. Sohl and Owens^112^ subdivided the Upper Cretaceous of Carolina coastal plain into three lithostratigraphic units, the Middendorf, Black Creek and Peedee Formations, raised the Black Creek to group status and subdivided this into three unconformity-bound formations, viz. in stratigraphic sequence, the Tar Heel, Bladen and Donoho Creek formations. Evidently, *Sabalites carolinensis* was recovered from what is now recognized as the Middendorf Formation, and a minimum age constraint can be established on the boundary between the Middendorf and Tar Heel Formations. The Middendorf is commonly considered Santonian in age, however, little material evidence has been presented in support of this, in part a consequent of the complex history of stratigraphic divisions at outcrop, in subsurface and offshore^105^. Habib and Miller^106^ established an age ‘not younger than Campanian’ on the basis of dinoflagellate biostratigraphy, but following the stratigraphic scheme outlined Campbell and Grohn^105^, the Middendorf is older that the Shepherd Grove Formation and, therefore, following the stratigraphy of Christopher and Prowell^107^, must be no younger than Santonian. Thus, we may established a minimum age constraint on the *Sabalites carolinesis* based on the Santonian-Campanian Boundary, coincident with the base of the *Scaphites leei III* Zone, dated to 83.64 Ma ± 0.23 Myr^67^, thus, 83.41 Ma.

**26. SG Musaceae | MRCA: *Musa-Oryza* | 74.6 Ma.**

**Fossil taxon and specimen.** *Spirematospermum chandlerae* has been described from isolated seeds and groups of seeds from the Neuse River locality, Black Creek Formation, southwest of Goldsboro, Wayne County, North Carolina, USA.

**Phylogenetic justification.** The phylogenetic relationships of this fossil have discussed in previous studies^103^.

**Minimum age.** 74.6 Ma.

**Age justification.** Reputedly Late Cretaceous (Early Campanian) in age^108^, the Black Creek Formation has been assigned to the *Exogyra ponderosa* Biozone which occurs beneath the *Didymoceras cheyennense Tethyan ammonoid biozone*^107^, the base of which is dated to 74.6 Ma^67^.

**27. SG Dioscoreales | MRCA: *Dioscorea-Colchicum* | 85.8 Ma.**

**Fossil taxon and specimen.** *Mabelia connatifila* [CUPC 1255: L. H. Bailey Hortorium Paleobotanical Collection,

Cornell University, Ithaca NY, USA] from the South Amboy Fire Clay Member of the Raritan Formation at the Old Crossman clay pit in Sayreville, New Jersey, USA^109^.

**Phylogenetic justification.** The phylogenetic assignment is based on the phylogenetic hypothesis reconstructed by Gandolfo et al.^109^.

**Minimum age.** 85.8 Ma.

**Age justification.** Clarke et al.^1^ argued that a minimum constraint on the age of this deposit could be established from Santonian-Campanian Boundary, however, Massoni et al.^80^ argue that a tighter correlation can be established with better rocks attributable to the CC13-14 Nannofossil zones in South Carolina, indicating a minimum age of 86.3 Ma ± 0.5 Myr, thus, 85.8 Ma.

**28. SG Oryzeae | MRCA *Oryza – Brachypodium |* 65.98 Ma.**

**Fossil taxon and specimen.** *Changii indicum* [Slide 13160, Q-14-3, Birbal Sahni I. Palaeobotany, Lucknow, India] from the Maastrichtian-Danian Deccan beds of India

**Phylogenetic justification.** The phylogenetic relationships of this fossil have discussed in previous studies^103^.

**Minimum age.** 65.98 Ma.

**Age justification.** We follow Iles *et al.*^103^ and their recommendation of the radiometric and magnetostrategraphic dating of the Deccan beds of India by Courtillot and Ren^110^ and the presence of dinosaur coprolites to be latest Maastrichtian, updated following Ogg & Hinnov^67^

**29. CG Eudicots | MRCA: *Escholzia-Capsella*| 119.6 Ma.**

**Fossil taxon and specimen**. *Hyrcantha decussata* [NJU-DES02001: Geological Institute, Chinese Academy of Sciences, Beijing], from the lower part of the Yixian Formation, Jehol Group, Liaoning Province, China^111^.

**Phylogenetic justification.** Similar to *Leefrutcus* from the Yixian formation of the Lower Cretaceous of China, *Hycantha* is considered to be a stem group representative of the Ranunculales^112^.

**Minimum age**. 119.6 Ma.

**Age justification.** The main fossil bearing beds have been dated and may be as old as 129.2 Ma^68^, however, in the absence of knowledge of the position of the fossils within the stratigraphy, relative to the sources of the absolute dates, a minimum age constraint can be derived from the Jiufontang Formation which overlies it. ^40^Ar/^39^Ar dating of a number of samples from the Jiufontang Formation has yielded an age of 120.3 ± 0.7 Ma for the volcanic tuffs^69^, establishing a minimum constraint of 119.6 Ma.

**30. CG Ericales core | MRCA: *Diospyros-Inula* | 85.8 Ma.**

**Fossil taxon and specimen.** *Paleoenkianthus sayrevillensis* [CUPC 1100: L. H. Bailey Hortorium, Cornell University, Ithaca NY, USA] from the South Amboy Fire Clay of the Raritan Formation, of which outcrops are exposed in the Old Crossman Clay Pit in Sayreville, New Jersey.

**Phylogenetic justification.** The phylogenetic relationships of this fossil has been tested based on morphological evidence^113^.

**Minimum age.** 85.8 Ma

**Age justification.** Clarke et al.^1^ argued that a minimum constraint on the age of this deposit could be established from Santonian-Campanian Boundary, however, Massoni et al.^80^ argue that a tighter correlation can be established with better rocks attributable to the CC13-14 Nannofossil zones in South Carolina, indicating a minimum age of 86.3 Ma ± 0.5 Myr, thus, 85.8 Ma.

**31. SG Asteraceae minus *Bernadesia* | MRCA: *Tanacetum - Inula* | 41.5 Ma.**

**Fossil taxon and specimen.** *Tubulifloridites antipodica* from onshore deposits taken from a paleochannel at Koingnaas, on the west coast of South Africa.

**Phylogenetic justification.** The newly described *Tubulifloridites lilliei* type A predates this estimates with an age of 76 – 66 Ma^114^ , however the assignment of this fossil and its affinity with Asteraceae remains controversial^115^. The placement of the pollen fossils of *T. antipodica* within Asteraceae minus *Bernadesia* is deemed reliable^116^.

**Minimum age.** 41.5 Ma.

**Age justification.** These occurrences are, described to occur alongside the planktic forams *Globigerinatheka index* and *Turborotalia centralis*^117^. *Globigerinatheka index* is known to range from 42.9 - 34.3 Ma^118^, but *Turborotalia centralis* is a junior synonym of *Turborotalia pomeroli*, which is known to range from 42.4-41.5 Ma^118^. Thus, the minimum age constraint on *Tubulifloridites antipodica* is 41.5 Ma.

**32. SG Myrtales | MRCA: *Eucalyptus-Capsella* | 83.3 Ma.**

**Fossil taxon and specimen.** *Esqueiria futabensis* [PP45419: Field Museum, Chicago IL, USA] from two levels in the Futaba Group exposed in Fukushima Prefecture. northeastern Honshu, Japan^119^.

**Phylogenetic justification.** The phylogenetic relationships have been established by several authors^120^.

**Minimum age.** 83.3 Ma.

**Age justification.** One locality, considered Coniacian, occurs in the Asamigawa Member of the Ashizawa Formation, on a tributary of the Kitaba River in Kamikitaba, Hirono-machi. Unfortunately, no material evidence has been presented to substantiate this age assignment (Takahashi et al.^119^, among others, merely cite the presence of unspecified Coniacian ammonites). The second locality is in the middle part of the Tamayama Formation, on the Kohisa River, Kohisa, Ouhisa machi, northeast of lwaki City. The Asamigawa Formation is the lowermost formation in the Futaba Group, and is overlain by the Kasamatsu Formation, in turn overlain by the Tamayama Formation. The age of the Tamayama Formation is substantiated on the presence of *Inoceramus amakusensis*^119^, which is restricted to the Santonian^121^. Thus, a minimum age constraint may be established on the Santonian-Campanian Boundary, dated as 83.6 Ma ± 0.3 Myr^67^, thus, 83.3 Ma.

**33. SG Sapindales | MRCA: *Citrus-Capsella* | 59.24 Ma.**

**Fossil taxon and specimen.** *Dipteronia brownii* [UF 15740E-23086: Florida Museum of Natural History, Gainesville FL, USA] from the Paleocene Fort Union Formation at Hell's Half Acre, Wyoming^122^.

**Phylogenetic justification.** This fossil is assigned to the extant genus *Dipteronia* which belongs to the subfamily Hippocantanoides of the family Sapindaceae. The extant genus is considered a Tertiary relict having two extant species endemic to China^123,124^. Being a possible stem group representative of the extant genus nested in the Sapindales provided the framework for this assignment.

**Minimum age.** 59.24 Ma.

**Age justification.** *Dipteronia brownii* occurs within the P4 Pollen Zone in the type section of Nichols and Ott^125^, which falls fully within Magnetic Anomaly Zone C26r^126^, the end of which is dated to 59.24 Ma in the combined age model of Vandenberghe et al.^127^.

**34. SG *Salicaceae* | MRCA: *Linum-Populus* | 48.57 Ma.**

**Fossil taxon and specimen.** *Pseudosalix handleyi* [UMNH PB-1: Utah Museum of Natural History,

Salt Lake City, USA] from lacustrine shales of the Parachute Creek Member of the Green River Formation in the vicinity of Bonanza, Utah, USA^128^.

**Phylogenetic justification.** Our node assignment follows the currently accepted interpretation of the fossil record of Salicaceae^129^.

**Minimum age.** 48.57 Ma.

**Age justification.** The Parachute Creek Member reaches into C22n magnetozone^130^, the minimum age of which can be established from the base of the succeeding C21r, dated to 48.57 Ma in the combined age model of Vandenberghe et al.^127^

1. Clarke, J., Donoghue, P. C. J. & Warnock, R. C. M. Establishing a timescale for plant evolution. *New Phytologist* **192**, 266-301 (2011).
2. Steemans, P. *et al.* Origin and Radiation of the Earliest Vascular Land Plants. *Science* **324**, 353-353, doi:10.1126/science.1169659 (2009).
3. Strother, P. K., Wood, G. D., Taylor, W. A. & Beck, J. H. Middle Cambriancryptospores and the origin of land plants. *Memoirs of the Association of Australasian Palaeontologists* **24**, 99–113 (2004).
4. Cooper, R. A. & Sadler, P. M. in *Geologic timescale 2012* (eds Felix M. Gradstein, James G. Ogg, Mark Schmitz, & Gabbi Ogg) 489-523 (Elsevier, 2012).
5. Peng, S., Babcock, L. E. & Cooper, R. A. in *Geologic timescale 2012* (eds Felix M. Gradstein, James G. Ogg, Mark Schmitz, & Gabbi Ogg) 437-488 (Elsevier, 2012).
6. Townrow, J.A. Two Triassic Bryophytes from South Africa. *South African Journal of Botany* **25**, 1-22 (1959).
7. Anderson, H.A. A review of the Bryophyta from the Upper Triassic Molteno Formation, Karoo Basin, South Africa. *Palaeontologica Africana* **19,** 21-30 (1976)
8. Anderson, J.M. & Anderson, H.M. The fossil content of the Upper Triassic Molteno Formation, South Africa. *Palaeontologica Africana* **25**, 39–59 (1984)
9. Ogg, J. G. in *The geologic time scale 2012* (eds F. M. Gradstein, J. G. Ogg, M. Schmitz, & G. Ogg) 681-730 (Elsevier, 2012).
10. Guo, C. Q., Edwards, D., Wu, P. C., Duckett, J. G., Hueber, F. M. & Li, C. S. *Riccardiothallus devonicus* gen. et sp. nov., the earliest simple thalloid liverwort from the Lower Devonian of Yunnan, China. *Review of Palaeobotany and Palynology* **176,** 35-40 (2012)
11. Hao, S. G., Gensel, P. G. & Wang, D. M. *Polythecophyton demissum*, gen.et sp. nov., a new plant from the Lower Devonian (Pragian) of Yunnan, China and its phytogeographic significance. *Review of Palaeobotany and Palynolog*y **116**, 55–71 (2001).
12. Becker, R. T., Gradstein, F. M. & Hammer, O. in *The geological timescale 2012* (eds F. M. Gradstein, J. G. Ogg, M. Schmitz, & G. Ogg) 559-601 (Elsevier, 2012).
13. Kenrick, P. & Crane, P. R. *The origin and early diversification of land plants: a cladistic study*. (Smithsonian Institution Press, 1997)
14. Zalasiewicz, J. A., Taylor, L., Rushton, A. W. A., Loydell, D. K., Rickards, R. B. & Williams, M. Graptolites in British stratigraphy. *Geological Magazine* **146**, 785-850 (2009).
15. Melchin, M. J., Sadler, P. M. & Cramer, B. D. in *Geologic timescale 2012* (eds Felix M. Gradstein, James G. Ogg, Mark Schmitz, & Gabbi Ogg) 525-558 (Elsevier, 2012).
16. De Souza, I. C. C., Branco, F. S. R. & Vargas, Y. L. Permian bryophytes of Western Gondwanaland from the Parana Basin in Brazil. *Palaeontology* **55,** 229-241 doi: 0.1111/j.1475-4983.2011.01111.x (2012).
17. Laenen, B. et al. (2014) Extant diversity of bryophytes emerged from successive post-Mesozoic diversification bursts. *Nature Communications* **5,** 6134, doi:10.1038/ncomms6134 (2014).
18. Santos, R. V., Souza, P. A., Alvarenga, C. J. S., Dantes, E. L., Pimentel, M. M., Oliveira, C. G. & Araújo, L. M. Shrimp U-Pb Zircon dating and Palinology of Bentonitic layers from Permian Irati Formation, Paraná Basin, Brazil*. Gondwana Research* **9**, 41– 65 doi:10.1016/j.gr.2005.12.001 (2006).
19. Davydov, V. I., Korn, D. & Schmitz, M. D. in *The geologic time scale 2012* (eds F. M. Gradstein, J. G. Ogg, M. Schmitz, & G. Ogg) 603-651 (Elsevier, 2012)
20. Kotyk, M. E., Basinger, J. F., Gensel, P. G. & de Freitas, T. A. Morphologically complex plant macrofossils from the Late Silurian of Arctic Canada. *American Journal of Botany* **89**, 1004-1013 (2002).
21. Wellman, C. H., Gensel, P. G. & Taylor, W. A. Spore wall ultrastructure in the early lycopsid *Leclercqia* (Protolepidodenrales) from the Lower Devonian of North America: evidence for a fundamental division in the lycopsids. *American Journal of Botany* **96**, 1849-1860, doi:10.3732/ajb.0800422 (2009).
22. Meyer-Berthaud, B., Fairon-Demaret, M., Steemans, P., Talent, J. & Gerrienne, P. (2003) The plant *Leclercqia* (Lycopsida) in Gondwana: implications for reconstructing Middle Devonian palaeogeography. *Geological Magazine* **140**, 119-130.
23. Magallon, S., Hilu, K. W. & Quandt, D. Land plant evolutionary timeline: gene effects are secondary to fossil constraints in relaxed clock estimation of age and substitution rates. *American Journal of Botany* **100**, 556-573 (2013).
24. Bonamo, P. M. (1977) *Rellimia Thomsonii* (Progymnospermopsida) from Middle Devonian of New York State. *American Journal of Botany* **64**, 1272-1285.
25. Skog, J. E. & Banks, H. P. (1973) *Ibyka amphikoma*, gen et sp-n - new protoarticulate precursor from late Middle Devonian of New York State. *American Journal of Botany* **60**, 366-380.
26. Bartholomew, A. J. & Brett, C. E. (2007) Correlation of Middle Devonian Hamilton Group-equivalent strata in east-central North America: implications for eustasy, tectonics and faunal provinciality. *Geological Society, London, Special Publications* **278**, 105-131.
27. Johnson, J. G., Klapper, G. & Sandberg, C. A. (1985) Devonian eustatic fluctuations in Euramerica. *Geological Society of America Bulletin* **96**, 567.
28. Fisher, D. W., Isachsen, Y. W., Rickard, L. V., Broughton, J. G. & Offield, T. W. *Geologic map of New York*. (New York State Museum Sciece Service, Geological Survey, 1962).
29. Rickard, L. V. *Correlation of the Devonian rocks in New York State. Map and Chart Series 4*. (New York State Musuem Science Service Geological Survey, 1964).
30. Klapper, G. in *Devonian biostratigraphy of New York, Part I.* (eds W. A. Oliver, Jr. & G. Klapper) 57-68 (IUGS SDS, 1981).
31. Kirchgasser, W. T. (2000) Correlation of stage boundaries in the Appalachian Devonian, Eastern United States. *Courier Forschungsinstitut Senckenberg* **225**, 271-284.
32. Pšenička, J. & Bek, J. Cuticles and spores of *Senftenbergia plumosa* (Artis) Bek and Pšenička from the Carboniferous of Pilsen Basin, Bohemian Massif. *Review of Palaeobotany and Palynology* **125**, 299-312 (2003)
33. Bek, J. & Pšenička, J. *Senftenbergia plumosa* (Artis) emend and their microspores from the Carboniferous of the Kladno and Pilsen. *Review of Palaeobotany and Palynology*. **116,** 213-232 (2001).
34. Schmidt, A. R., Heinrichs, J. & Schneider, H. Burmese amber fossils bridge the gap in the Cretaceous record of polypod ferns. *Perspectives in Plant Ecology, Evolution and Systematics* **18**, 70-78 (2016)
35. Cruickshank, R. D. & Ko, K. Geology of an amber locality in the Hukawng valley, Northern Myanmar. *Journal of Asian Earth Sciences* **21**, 441–445 (2003).
36. Shi, G. H., Grimaldi, D. A., Harlow, G. E., Wang, J., Yang, M. C., Lei, W. Y., Li, Q. L. & Li, X. H. Age constraint on Burmese amber based on U-Pb dating of zircons. *Cretaceous Researc*h **37,** 155–163 (2012).
37. Trivett, M. L. Growth architecture, structure, and relationships of *Cordaixylon iowensis* nov comb (Cordaitales). *International Journal of Plant Sciences* **153**, 273-287 (2002)
38. Janousek, T. J. & Pope, J. P. Petrology, petrography and conodont biostratigraphy of the Laddsdale Coal interval, along Whitebreast Creek, Bauer, Iowa. *GSA North-Central Section, 48th Annual Meeting, Abstracts* **16-5** (2014)
39. Heckel, P. H. Pennsylvanian stratigraphy of Northern Midcontinent Shelf and biostratigraphic correlation of cyclothems. *Stratigraphy* **10**, 3-39 (2013)
40. Barrick, J. E., Lambert, L. L., Heckel, P. H., Rosscoe, S. J. & Boardman, D. R. Midcontinent Pennsylvanian conodont zonation. *Stratigraphy* **10**: 55-72 (2013).
41. Prestianni, C. Early diversification of seeds and seed-like structures. *Carnets De Geologie*, 33-38 (2003)
42. Rothwell, G. W., Scheckler, S. E. & Gillespie, W. H. *Elkinsia* gen nov, a late Devonian gymnospermn with cupulate ovules. *Botanical Gazette* **150**: 170-189 (1989).
43. Streel, M. & Scheckler, S. E. Miospore lateral distribution in upper Fammenian alluvial lagoonal to tidal facies from eastern United States and Belgium. *Review of Palaeobotany and Palynology* **64**, 315-324 (1990).
44. Streel, M., Higgs, K., Loboziak, S., Riegel, W. & Steemans, P. Spore stratigraphy and correlation with faunas and floras in the type marine Devonian of the Ardenne-Rhenish regions. *Review of Palaeobotany and Palynology* **50**, 211-229 (1987).
45. House, M. R. & Gradstein, F. M. in *A geologic timescale 2004* (eds F. M. Gradstein, J. G. Ogg, & A. G. Smith) 202-221 (Cambridge University Press, 2004).
46. Zanne, A. E. *et al.* Three keys to the radiation of angiosperms into freezing environments. *Nature* **506**, 89-92, (2014).
47. Gao, Z. & Thomas, B. A. A review of fossil cycad megasporophylls, with new evidence of Crossozamia pomel and its associated leaves from the lower Permian of Taiyuan, China. *Review of Palaeobotany and Palynology* **60**, 205-223 (1989).
48. Nagalingum, N. S. *et al.* Recent synchronous radiation of a living fossil. *Science* **334**, 796-799, doi:10.1126/science.1209926 (2011).
49. Hermsen, E. J., Taylor, T. N., Taylor, E. L. & Stevenson, D. W. Cataphylls of the Middle Triassic cycad Antarcticycas schopfii and new insights into cycad evolution. *American Journal of Botany* **93**, 724-738 (2006).
50. Wang, J. Late Paleozoic macrofloral assemblages from Weibei Coalfield, with reference to vegetational change through the Late Paleozoic Ice-age in the North China Block. *International Journal of Coal Geology* **83**, 292-317, doi:10.1016/j.coal.2009.10.007 (2010).
51. Henderson, C. M., Gradstein, F. M. & Hammer, O. in *The geologic timescale 2012* (eds F. M. Gradstein, J. G. Ogg, M. Schmitz, & G. Ogg) 653-679 (Elsevier, 2012).
52. Wieland, G. W. The Cerro Cuadrado petrified forest. *Carnegie Institution of Washington Publication* **449**, 1-183 (1935).
53. Calder, M. G. A coniferous petrified forest in Patagonia. *Bulletin of the British Museum (Natural History): Geology* **2**, 99-138 (1953).
54. Stockey, R. A. Seeds and embryos of *Araucaria mirabilis*. *American Journal of Botany* **62**, 856-868 (1975).
55. Stockey, R. A. Reproductive biology of Cerro Cuadrado fossil conifers: Ontogeny and reproductive strategies in *Araucaria mirabilis* (Spegazzini) Windhausen. *Palaeontographica Abteilung B* **166**, 1-15 (1978).
56. Wilde, M. H. & Eames, A. J. The ovule and seed of *Araucaria badwillii* with discussion of the taxonomy of the genus. 1. Morphology. *Annals of Botany* **12**, 311-& (1948).
57. Spalleti, L., Iñiguez Rodríguez, A. M. & Masón, M. Edades radimétricas de piroclastitas y volvanitas del Grupo Bahía Laura, Gran Bajo de San Julián, Santa Cruz. *Revista de la Asociación Geológica Argentina* **37**, 483-485 (1982).
58. Florin, R. Evolution in cordaites and conifers. *Acta Horti Bergiani* **15**, 285-388 (1951).
59. Yao, X. L., Taylor, T. N. & Taylor, E. L. A taxodiaceous seed cone from the Triassic of Antarctica. *American Journal of Botany* **84**, 343-354 (1997).
60. Axsmith, B. J., Taylor, T. N. & Taylor, E. L. Anatomically preserved leaves of the conifer Notophytum krauselii (Podocarpaceae) from the Triassic of Antarctica. *American Journal of Botany* **85**, 704-713 (1998).
61. Krassilov, V. A. New floral structure from the Lower Cretaceous of Lake Baikal Area. *Review of Palaeobotany and Palynology* **47**, 9-16 (1986)
62. Godefroit, P. *Bernissart dinosaurs and Early Cretaceous terrestrial ecosystems*. (Indiana University Press, 2012).
63. Vakhrameev, V. & Kotova, I. Ancient angiosperms and accompanying plants from the Lower Cretaceous of Transbaikalia. *Paleontological Journal* **4**, 487-495 (1977).
64. Vakhrameev, V. *Jurassic and Cretaceous floras and climates of the Earth*. (Cambridge University Press, 1991).
65. Chen, P. *et al.* Jianshangou Bed of the Yixian Formation in West Liaoning, China. *Science in China Series D: Earth Sciences* **48**, 298-312, doi:10.1360/04yd0038 (2005).
66. Dettmann, M. E. & Thomson, M. R. A. Cretaceous palynomorphs from the James-Ross Island area, Antarctica - a pilot-study. *British Antarctic Survey Bulletin* **77**, 13-59 (1987).
67. Ogg, J. G. & Hinnov, L. A. in *The geologic time scale 2012* Vol. 2 (eds F. M. Gradstein, J. G. Ogg, M. Schmitz, & G. Ogg) 793-853 (Elsevier, 2012).
68. Chang, S.-C., Zhang, H., Hemming, S. R., Mesko, G. T. & Fang, Y. Chronological evidence for extension of the Jehol Biota into Southern China. *Palaeogeography, Palaeoclimatology, Palaeoecology* **344–345**, 1-5, doi:10.1016.2012.05.014 (2012).
69. He, H. Y. *et al.* Timing of the Jiufotang Formation (Jehol Group) in Liaoning, northeastern China, and its implications. *Geophysical Research Letters* **31**, 1-4 (2004).
70. Hughes, N. F. & McDougall, A. B. Barremian-Aptian angiospermid pollen records from southern England. *Review of Palaeobotany and Palynology* **65**, 145-151 (1990).
71. Judd, W. S. & Olmstead, R. G. A survey of tricolpate (eudicot) phylogenetic relationships. *American Journal of Botany* **91**, 1627-1644 (2004).
72. Liu, Z. J. & Wang, X. A perfect flower from the Jurassic of China. *Hist Biol* **28**, 707-719, doi:10.1080/08912963.2015.1020423 (2016).
73. Han, G. *et al.* A whole plant herbaceous angiosperm from the Middle Jurassic of China. *Acta Geologica Sinica* **90**, 19-29 (2016).
74. Liu, Z.-J. & Wang, X. Yuhania: a unique angiosperm from the Middle Jurassic of Inner Mongolia, China. *Historical Biology*, 1-11, doi:10.1080/08912963.2016.1178740 (2016).
75. Mohr, B. A. R., Bernardes-De-Oliveira, M. E. C. & Taylor, D. W. Pluricarpellatia, a nymphaealean angiosperm from the Lower Cretaceous of northern Gondwana (Crato Formation, Brazil). *Taxon* **57**, 1147-1158 (2008).
76. Taylor, D. W., Brenner, G. J. & Basha, S. H. Scutifolium jordanicum gen. et sp nov (Cabombaceae), an aquatic fossil plant from the Lower Cretaceous of Jordan, and the relationships of related leaf fossils to living genera. *American Journal of Botany* **95**, 340-352 (2008).
77. Batten, D. J. in *The Crato fossil beds of Brazil - window into an ancient world* (eds D. M. Martill, G. Bechly, & R. F. Loveridge) 566-573 (Cambridge University Press, 2007).
78. Martill, D. M. The age of the Cretaceous Santana Formation fossil Konservat Lagerstatten of north-east Brazil: a historical review and an appraisal of the biochronostratigraphic utility of its palaeobiota. *Cretaceous Research* **28**, 895-920, doi:10.1016/j.cretres.2007.01.002 (2007).
79. Heimhofer, U. & Hochuli, P.-A. Early Cretaceous angiosperm pollen from a low-latitude succession (Araripe Basin, NE Brazil). *Review of Palaeobotany & Palynology* **161**, 105-126, doi:10.1016/j.revpalbo.2010.03.010 (2010).
80. Massoni, J., Doyle, J. A. & Sauquet, H. Fossil calibration of Magnoliidae, an ancient lineage of angiosperms. *Palaeontologica Electronica* (2014).
81. Mohr, B. A. R., Coiffard, C. & Bernardes-de-Oliveira, M. E. C. Schenkeriphyllum glanduliferum, a new magnolialean angiosperm from the Early Cretaceous of Northern Gondwana and its relationships to fossil and modern Magnoliales. *Review of Palaeobotany and Palynology* **189**, 57-72, doi:10.1016/j.revpalbo.2012.08.004 (2013).
82. Friis, E. M., Pedersen, K. R. & Crane, P. R. Fossil evidence of water lilies (Nymphaeales) in the Early Cretaceous. *Nature* **410**, 357-360 (2001).
83. Friis, E. M., Pedersen, K. R., Von Balthazar, M., Grimm, G. W. & Crane, P. R. *Monetianthus mirus* gen. et sp. nov., a nymphaealean flowers from the Early Cretaceous of Portugal. *International Journal of Plant Science* **170**, 1086-1101 (2009).
84. Smith, S. A., Beaulieu, J. M. & Donoghue, M. J. An uncorrelated relaxed-clock analysis suggests an earlier origin for flowering plants. *Proceedings of the National Academy of Sciences* **107**, 5897-5902, doi:10.1073/pnas.1001225107 (2010).
85. Amireh, B. S., Jarrar, G., Henjes-Kunst, F. & Schneider, W. K-Ar dating, X-ray diffractometry, optical and scanning electron microscopy of glauconites from the Early Cretaceous Kurnub. *Geological Journal* **33**, 49-65 (1998).
86. Friis, E.M., Crane, P.R. & Pedersen, K.R*. Anacostia*, a new basal angiosperm from the Early Cretaceous of North America and Portugal with trichotomocolpate/monocolpate pollen. *Grana* **36**, 225-244, doi:10.1080/00173139709362611, (1997).
87. Doyle, J. A., Endress, P. K. & Upchurch, G. R., Jr. Early Cretaceous monocots: a phylogenetic evaluation. *Sbornik Narodniho Muzea v Praze Rada B Prirodni Vedy* **64**, 59-87 (2008).
88. Doyle, J. A. & Robbins, E. I. Angiosperm pollen zonation of the continental Cretaceous of the Atlantic coastal plain and its application to deep wells in the Salisbury Embayment. *Palynology*, 43-78 (1977).
89. Doyle, J. A. Recognising angiosperm clades in the Early Cretaceous fossil record. *Historical Biology* **27**, 414-429, doi:10.1080/08912963.2014.938235 (2015).
90. Doyle, J. A. & Endress, P. K. Integrating Early Cretaceous Fossils into the Phylogeny of Living Angiosperms: ANITA Lines and Relatives of Chloranthaceae. *International Journal of Plant Sciences* **175**, 555-600, doi:10.1086/675935 (2014).
91. Mohr, B. A. R. & Bernardes-de-Oliveira, M. E. C. Endressinia brasiliana, a magnolialean angiosperm from the lower Cretaceous Crato Formation (Brazil). *International Journal of Plant Sciences* **165**, 1121-1133 (2004).
92. Mohr, B. A. R., Coiffard, C. & Bernardes-de-Oliveira, M. E. C. Schenkeriphyllum glanduliferum, a new magnolialean angiosperm from the Early Cretaceous of Northern Gondwana and its relationships to fossil and modern Magnoliales. *Review of Palaeobotany and Palynology* **189**, 57-72, doi:10.1016/j.revpalbo.2012.08.004 (2013).
93. Sauquet, H. *et al.* Phylogenetic analysis of Magnoliales and Myristicaceae based on multiple data sets: implications for character evolution. *Botanical Journal of the Linnean Society* **142**, 125-186 (2003).
94. Smith, S. Y. & Stockey, R. A. Establishing a fossil record for the perianthless Piperales: *Saururus tuckerae* sp. nov. (Saururaaceae) from the Middle Eocene Princeton Chert. *American Journal of Botany* **94**, 1642–1657 (2007).
95. Rouse, G. E. & Mathews, W. H. Radioactive dating of Tertiary plant-bearing deposits. *Science* **133**, 1079-1080 (1961).
96. Mathews, W. H. Potassium-argon age determinations of Cenozoic volcanic rocks from British Columbia. *Geological Society of America Bulletin* **75**, 465-468 (1964).
97. Hills, L. V. & Baadsgaard, H. Potassium-argon dating of some Lower Tertiary strata in British Columbia. *Bulletin of Canadian Petroleum Geology* **15**, 138-149 (1967).
98. Read, P. B. Geology and industrial minerals of the Tertiary basins, south-central British Columbia. *British Columbia Geological Survey Geo-File* **2000** (2000).
99. Moss, P. T., Greenwood, D. R. & Archibald, S. B. Regional and local vegetation community dynamics of the Eocene Okanagan Highlands (British Columbia – Washington State) from palynology. *Canadian Journal of Earth Sciences* **42**, 187-204 (2005).
100. Hochuli, P. A., Heimhofer, U. & Weissert, H. Timing of early angiosperm radiation: recalibrating the classical succession. *Journal of the Geological Society* **163**, 587-594 (2006).
101. Hughes, N. F. *The enigma of angiosperm origins*. (Cambridge University Press, 1994).
102. Berry, E. W. The Upper Cretaceous and Eocene floras of South Carolina and Georgia. *United States Geological Survey Professional Paper* **84**, 1-200 (1914).
103. Hertweck, K. L. *et al.* Phylogenetics, divergence times and diversification from three genomic partitions in monocots. *Botanical Journal of the Linnean Society* **178**, 375-393, doi:10.1111/boj.12260 (2015).
104. Iles, W. J. D., Smith, S. Y., Gandolfo, M. A. & Graham, S. W. Monocot fossils suitable for molecular dating analyses. *Botanical Journal of the Linnean Society* **178**, 346-374, doi:10.1111/boj.12233 (2015).
105. Campbell, B. G. & Gohn, G. S. Stratigraphic framework for geologic and geohydrologic studies of the subsurface Cretaceous section near Charleston, South Carolina. *United States Geological Survey Map MF-2273*, 1-11 (1994).
106. Habib, D. & Miller, J. A. Dinoflagellate species and organic facies evidence of marine transgression and regression in the atlantic coastal plain. *Palaeogeography, Palaeoclimatology, Palaeoecology* **74**, 23-47, doi:10.1016/0031-0182(89)90018-7 (1989).
107. Christopher, R. A. & Prowell, D. C. A palynological biozonation for the uppermost Santonian and Campanian Stages (Upper Cretaceous) of South Carolina, USA. *Cretaceous Research* **31**, 101-129, doi:10.1016/j.cretres.2009.09.004 (2010).
108. Friis, E. M. *Spirematospermum chandlerae* sp. nov., an extinct species of Zingiberacea from the North American Cretaceous. *Tertiary Research* **9**, 7-12 (1988).
109. Gandolfo, M. A., Nixon, K. C. & Crepet, W. L. Triuridaceae fossil flowers from the Upper Cretaceous of New Jersey. *American Journal of Botany* **89**, 1940-1957 (2002).
110. Courtillot, V. E. & Renne, P. R. On the ages of flood basalt events. Comptes Rendus Geoscience 335, 113–140, doi:10.1016/S1631-0713(03)00006-3, (2003).
111. Dilcher, D. L., Sun, G., Ji, Q. & Li, H. Q. An early infructescence Hyrcantha decussata (comb. nov.) from the Yixian Formation in northeastern China. *Proceedings of the National Academy of Sciences of the United States of America* **104**, 9370-9374, doi:10.1073/pnas.0703497104 (2007)
112. Wang, W., Dilcher, D. L., Sun, G., Wang, H.-S. & Chen, Z.-D. Accelerated evolution of early angiosperms: Evidence from ranunculalean phylogeny by integrating living and fossil data. *Journal of Systematics and Evolution* **54**, 336-341, doi:10.1111/jse.12090 (2016).
113. Crepet, W. L., Nixon, K. C. & Daghlian, C. P. Fossil Ericales from the Upper Cretceous of New Jersey. *International Journal of Plant Sciences* **174**, 572-584 (2012).
114. Barreda, V. D., Palazzesi, L., Tellería, M. C., Olivero, E. B., Raine, J. I. & Forest, F. Early evolution in the angiosperm clade Asteraceae in the Cretaceous of Antarctica. *Proceedings of the National Academy of Sciences USA* **112,** 10989-10994 doi: 10.1073/pnas.1423653112, (2015).
115. Panero, J. L. Phylogenetic uncertainty and fossil calibration of Asteraceae chronograms. *Proceedings of the National Academy of Sciences USA* **113,** E411, doi: 10.1073/pnas.1517649113, (2016).
116. Martínez-Millán, M. Fossil record and age of the Asteridae. *Botanical Review* **76**, 83-135 (2010).
117. Zavada, M. & de Villiers, S. Pollen of the Asteraceae from the Paleocene-Eocene of South Africa. *Grana* **39**, 39-45, doi:10.1080/00173130150503795 (2000).
118. Wade, B. S., Pearson, P. N., Berggren, W. A. & Pälike, H. Review and revision of Cenozoic tropical planktonic foraminiferal biostratigraphy and calibration to the geomagnetic polarity and astronomical time scale. *Earth-Science Reviews* **104**, 111-142, doi:10.1016/.2010.09.003, (2011).
119. Takahashi, M., Crane, P. R. & Ando, H. *Esgueiria futabensis* sp. nov., a new angiosperm flower from the Upper Cretaceous (lower Coniacian) of northeastern Honshu, Japan. *Paleontological Research* **3**, 81-87 (1999).
120. Friis, E. M., Pedersen, K. R. & Crane, P. R. Cretaceous angiosperm flowers: Innovation and evolution in plant reproduction. *Palaeogeography, Palaeoclimatology, Palaeoecology* **232**, 251-293 (2006).
121. Yazykova, E. Ammonite and inoceramid radiations after the Santonian–Campanian bioevent in Sakhalin, Far East Russia. *Lethaia* **35**, 51-60 (2002).
122. McClain, A. M. & Manchester, S. R. Dipteronia (Sapindaceae) from the Tertiary of North America and Implications for the Phytogeographic History of the Aceroideae. *American Journal of Botany* **88**, 1316-1325, doi:10.2307/3558343 (2001).
123. Qiu, Y. L. *et al.* A nonflowering land plant phylogeny inferred from nucleotide sequences of seven chloroplast, mitochondrial, and nuclear genes. *International Journal of Plant Sciences* **168**, 691-708 (2007).
124. Manchester, S. R., Chen, Z.-D., Lu, A.-M. & Uemura, K. Eastern Asian endemic seed plant genera and their paleogeographic history throughout the Northern Hemisphere. *Journal of Systematics and Evolution* **47**, 1-42, doi:10.1111/j.1759-6831.2009.00001.x (2009).
125. Nichols, D. J. & Ott, H. L. Biostratigraphy and evolution of the *Momipites-Caryapollenites* lineage in the early Tertiary in the Wind River Basin, Wyoming. *Palynology* **2**, 93-112 (1978).
126. Peppe, D. J. Megafloral change in the early and middle Paleocene in the Williston Basin, North Dakota, USA. *Palaeogeography, Palaeoclimatology, Palaeoecology* **298**, 224-234, doi:10.1016/j.palaeo.2010.09.027 (2010).
127. Vandenberghe, N., Hilgen, F. J. & Speijer, R. P. in *The geologic timescale 2012* (eds F. M. Gradstein, J. G. Ogg, M. Schmitz, & G. Ogg) 855-921 (Elsevier, 2012).
128. Boucher, L. D., Manchester, S. R. & Judd, W. S. An extinct genus of Salicaceae based on twigs with attached flowers, fruits, and foliage from the Eocene Green River Formation of Utah and Colorado, USA. *American Journal of Botany* **90**, 1389-1399 (2003).
129. Manchester, S. R., Judd, W. S. & Handley, B. Foliage and fruits of early poplars (Salicaceae: Populus) from the Eocene of Utah, Colorado, and Wyoming. *International Journal of Plant Sciences* **167**, 897-908, doi:10.1086/503918 (2006).
130. Smith, M. E., Carroll, A. R. & Singer, B. S. Synoptic revision of a major ancient lake system: Eocene Green River Formation, western United States. *GSA Bulletin* **120**, 54-84, doi:10.1130/B26073.1 (2008).

**Supplementary Information S3.** The 33 gene families containing a clear signal of the ζ and ε duplication, or both. Referenced according to their orthogroups on Plaza 3.0 and the original study of Jiao et al.

| Gene Family | Plaza 3.0 Orthogroup | Jiao *et al.* 2011 orthogroup | Taxon coverage | Amino acid alignment length |
| --- | --- | --- | --- | --- |
| Cyclin-dependent kinase (CDK) | ORTHO03D000012 | 174 | 249 | 1215 |
| Phytochrome | ORTHO03D000373 | 361 | 231 | 967 |
| Homeobox leucine zipper | HOMO03D000716 | 245 | 242 | 672 |
| NAF domain kinase | ORTHO03D000089 | 385 | 204 | 515 |
| Serine/threonine protein kinase | ORTHO03D000280 | 477 | 231 | 544 |
| WD-40 repeat domain | ORTHO03D000598 | 576 | 220 | 588 |
| Trehalose phosphatase | [ORTHO03D000066](http://bioinformatics.psb.ugent.be/plaza/versions/plaza_v3_dicots/gene_families/view/ORTHO03D000066) | 170 | 132 | 139 |
| Actinin-type acting binding protein | ORTHO03D000306 | 493 | 116 | 513 |
| Oligouridylate binding proetin | [ORTHO03D000455](http://bioinformatics.psb.ugent.be/plaza/versions/plaza_v3_dicots/gene_families/view/ORTHO03D000455) | 1467 | 96 | 547 |
| Protein phosphatase 2A regulatory B | [ORTHO03D000116](http://bioinformatics.psb.ugent.be/plaza/versions/plaza_v3_dicots/gene_families/view/ORTHO03D000116) | 231 | 87 | 341 |
| Glucose-6-phosphate-1 dehydrogenase | [ORTHO03D001663](http://bioinformatics.psb.ugent.be/plaza/versions/plaza_v3_dicots/gene_families/view/ORTHO03D001663) | 880 | 110 | 333 |
| CDPK related kinase | [ORTHO03D000082](http://bioinformatics.psb.ugent.be/plaza/versions/plaza_v3_dicots/gene_families/view/ORTHO03D000082) | 215 | 122 | 383 |
| Protein phosphatase 2C | [ORTHO03D010475](http://bioinformatics.psb.ugent.be/plaza/versions/plaza_v3_dicots/gene_families/view/ORTHO03D010475) | 1692 | 104 | 215 |
| ATP-citrate lyase | [ORTHO03D000577](http://bioinformatics.psb.ugent.be/plaza/versions/plaza_v3_dicots/gene_families/view/ORTHO03D000577) | 1045 | 119 | 420 |
| Inositol transporter | [ORTHO03D000623](http://bioinformatics.psb.ugent.be/plaza/versions/plaza_v3_dicots/gene_families/view/ORTHO03D000623) | 542 | 99 | 365 |
| NADPH Reductase | [ORTHO03D000526](http://bioinformatics.psb.ugent.be/plaza/versions/plaza_v3_dicots/gene_families/view/ORTHO03D000526) | 658 | 108 | 460 |
| RAB GDI protein | [ORTHO03D000565](http://bioinformatics.psb.ugent.be/plaza/versions/plaza_v3_dicots/gene_families/view/ORTHO03D000565) | 606 | 113 | 443 |
| D-3-phosphoglycerate dehydrogenase | [ORTHO03D001648](http://bioinformatics.psb.ugent.be/plaza/versions/plaza_v3_dicots/gene_families/view/ORTHO03D001648) | 572 | 108 | 462 |
| Tetratricopeptide repeat | [ORTHO03D000272](http://bioinformatics.psb.ugent.be/plaza/versions/plaza_v3_dicots/gene_families/view/ORTHO03D000272) | 601 | 126 | 410 |
| GRPE nucleotide exchange factor | [ORTHO03D000565](http://bioinformatics.psb.ugent.be/plaza/versions/plaza_v3_dicots/gene_families/view/ORTHO03D000565) | 2062 | 136 | 176 |
| Mitochondrial substrate carrier | [HOM03D002662](http://bioinformatics.psb.ugent.be/plaza/versions/plaza_v3_dicots/gene_families/view/HOM03D002662) | 2590 | 132 | 228 |
| Heatshock protein | [ORTHO03D000072](http://bioinformatics.psb.ugent.be/plaza/versions/plaza_v3_dicots/gene_families/view/ORTHO03D000072) | 1276 | 139 | 441 |
| WD40 repeat family protein | [ORTHO03D009815](http://bioinformatics.psb.ugent.be/plaza/versions/plaza_v3_dicots/gene_families/view/ORTHO03D009815) | 1852 | 154 | 248 |
| DEAD-box ATP dependent helicase | [ORTHO03D000937](http://bioinformatics.psb.ugent.be/plaza/versions/plaza_v3_dicots/gene_families/view/ORTHO03D000937) | 1711 | 129 | 496 |
| Unknown function DUF 292 | [ORTHO03D003832](http://bioinformatics.psb.ugent.be/plaza/versions/plaza_v3_dicots/gene_families/view/ORTHO03D003832) | 1845 | 132 | 160 |
| Vacuolar Sorting Receptor | [ORTHO03D000149](http://bioinformatics.psb.ugent.be/plaza/versions/plaza_v3_dicots/gene_families/view/ORTHO03D000149) | 262 | 148 | 482 |
| Glycoside hydrolase family 31 | [ORTHO03D000220](http://bioinformatics.psb.ugent.be/plaza/versions/plaza_v3_dicots/gene_families/view/ORTHO03D000220) | 427 | 141 | 621 |
| Phospholipase C family | [ORTHO03D000189](http://bioinformatics.psb.ugent.be/plaza/versions/plaza_v3_dicots/gene_families/view/ORTHO03D000189) | 553 | 121 | 314 |
| Alanine glyoxalate transaminase | [ORTHO03D000974](http://bioinformatics.psb.ugent.be/plaza/versions/plaza_v3_dicots/gene_families/view/ORTHO03D000974) | 900 | 131 | 436 |
| Nramp2 family | [ORTHO03D000433](http://bioinformatics.psb.ugent.be/plaza/versions/plaza_v3_dicots/gene_families/view/ORTHO03D000433) | 571 | 106 | 376 |
| Long chain fatty acid coenzyme A | [ORTHO03D000603](http://bioinformatics.psb.ugent.be/plaza/versions/plaza_v3_dicots/gene_families/view/ORTHO03D000603) | 745 | 143 | 646 |
| Long-chain acyl-CoA synthetase 3 | [ORTHO03D000292](http://bioinformatics.psb.ugent.be/plaza/versions/plaza_v3_dicots/gene_families/view/ORTHO03D000292) | 384 | 167 | 647 |
| Vacuolar Sorting Receptor | ORTHO03D000149 | 262 | 168 | 483 |
